# CD63, MHC class 1, and CD47 identify subsets of extracellular vesicles containing distinct populations of noncoding RNAs

**DOI:** 10.1038/s41598-018-20936-7

**Published:** 2018-02-07

**Authors:** Sukhbir Kaur, Abdel G. Elkahloun, Anush Arakelyan, Lynn Young, Timothy G. Myers, Francisco Otaizo-Carrasquero, Weiwei Wu, Leonid Margolis, David D. Roberts

**Affiliations:** 1 Laboratory of Pathology, Center for Cancer Research, National Cancer Institute, National Institutes of Health, Bethesda, MD 20982 USA; 2 Cancer Genetics Branch, National Human Genome Research Institute, National Institutes of Health, Bethesda, MD 20892 USA; 3 Section of Intercellular Interactions, Eunice Kennedy-Shriver National Institute of Child Health and Human Development, National Institutes of Health, Bethesda, MD 20982 USA; 4 National Institutes of Health Library, Division of Library Services, Office of Research Services, National Institutes of Health, Bethesda, MD 20892 USA; 5 Genomic Technologies Section, Research Technologies Branch, National Institute of Allergy and Infectious Diseases, National Institutes of Health, Bethesda, MD 20892 USA

## Abstract

Extracellular vesicles (EVs) mediate the intercellular transfer of RNAs, which alter gene expression in target cells. EV heterogeneity has limited progress towards defining their physiological functions and utility as disease-specific biomarkers. CD63 and MHC1 are widely used as markers to purify EVs. CD47 is also present on EVs and alters their effects on target cells, suggesting that specific surface markers define functionally distinct EVs. This hypothesis was addressed by comparing Jurkat T cell EVs captured using CD47, CD63, and MHC1 antibodies. These EV subsets have similar sizes but divergent RNA contents. Apart from differences in numbers of nonannotated transcripts, CD63^+^, MHC1^+^, and CD47^+^ EVs have similar overall contents of most noncoding RNA classes, but the relative enrichment of specific RNAs differs. The enrichment of micro-RNAs is highly divergent, and some including miR320a are selectively concentrated in CD47^+^ EVs. Small nucleolar RNAs including SNORD116@ and SNHG10 are also selectively enriched in CD47^+^ EVs, whereas no small nuclear RNAs are enriched in CD47^+^ EVs. Conversely, MHC1^+^ EVs are selectively enriched in a subset of tRNAs including TRE-CTC and TRR-CCG. This heterogeneity in RNA composition suggests multiple sorting mechanisms that direct specific RNAs into subsets of EVs that express specific surface markers.

## Introduction

Ongoing investigations of extracellular vesicles (EVs) are revealing diverse and complex functions in cell-cell communication, mediated in part by their role in the intercellular transfer of RNAs^1–3^. The presence of disease-associated EVs in biological fluids such as saliva, urine, cerebrospinal fluid, and blood^4,5^ is providing a new source for biomarkers for diseases including cancer^6^, osteo- and rheumatoid arthritis^7^, and neurodegenerative disorders^8^. Engineered EVs are also promising deliver vehicles for therapeutic uses including the targeted delivery of miRNAs^9,10^.

To achieve these goals, barriers must be overcome to standardize EV nomenclature^11^, characterize their heterogeneity^12^, and define the molecular mechanisms controlling passive or active sorting of specific RNAs into different types of EVs^13^. To investigate the mechanisms of EV biogenesis and RNA sorting from their parent cells it is also important to consider biases introduced by specific methods for isolation and processing of EVs^14,15^. To date no standard method has been established for isolation of EVs. Various surface markers of EVs have been identified. Antibodies to CD9, CD63, CD81, and MHC class 1 (MHC1) have been used for immunoaffinity purification of EVs bearing these membrane proteins^16–18^, but the efficiency of these capture methods is not well documented and requires optimization of the ratio of magnetic particles versus EVs. Furthermore, it is unclear whether EVs produced by the same cell but lacking these markers differ in their RNA content or functional activity.

We and others have reported that CD47 is also present on EVs^19–21^. We found that T cell-derived EVs alter gene expression and functional signaling in endothelial cells in a CD47-dependent manner^21^. To further characterize EVs that express CD47 we have examined its expression on EVs captured using antibodies recognizing the established markers CD63 and MHC1. We also isolated subsets of EVs lacking or expressing each of these proteins and analyzed their small RNA contents using next generation sequencing. We report here that each marker-defined subset of EVs has a distinct RNA profile and is enriched in different miRNAs and other coding and noncoding RNAs relative to EVs lacking each respective marker. This suggests the existence of multiple sorting pathways that package RNAs into distinct EV populations within the same cell.

## Results

### Size Characterization and distribution of CD47^+^ EVs

**Figure 1:**
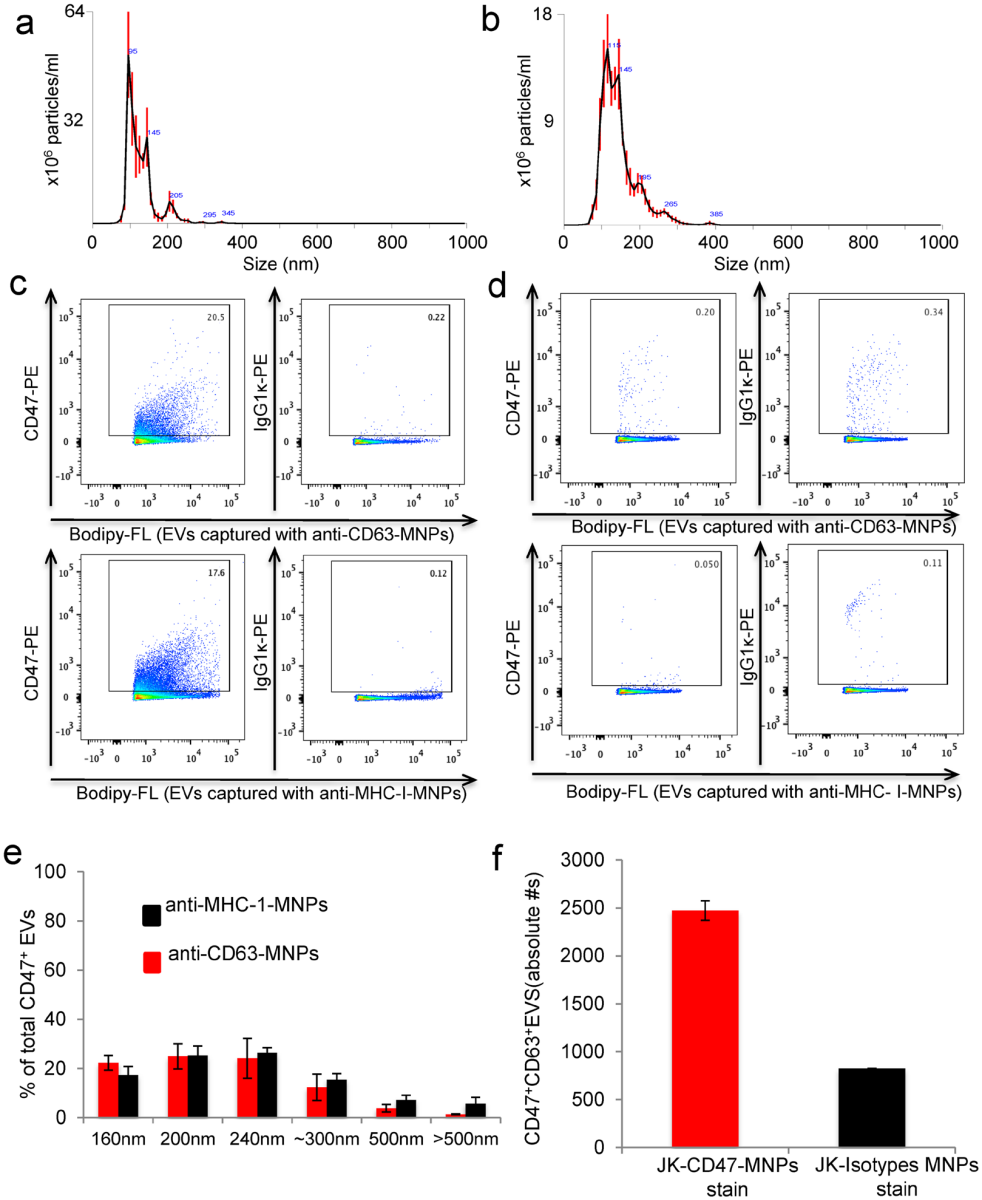
Characterization of Jurkat T cell EV fractions and CD47 expression. **(a,b)** EVs were extracted from wild type (**a**) and CD47-deficient Jurkat T cells (**b**) using the Exo-Quick kit, and vesicle size and concentration were quantified by Nanosight analysis. (**c)** EVs released by Jurkat cells were labeled using Bodipy-FL and captured with anti-CD63-MNPs (upper panel) or with anti-MHC I-MNPs (lower panel) and stained with PE-conjugated anti-CD47 or isotype control antibodies. Representative experiment out of 3. (**d)** EVs released by CD47-deficient JinB8 cells were captured with anti-CD63-MNPs (upper panel) or with anti-MHC I-MNPs (lower panel) and stained with anti-CD47 or with isotype control antibodies. Representative experiment of out of 3. (**e**) Size distribution of CD47^+^ EVs captured with anti-CD63-MNPs (red bars) or with anti-MHC1-MNPs (black bars). Representative experiment out of 3. **(f)** EVs released by Jurkat cells were captured with anti-CD47-MNPs and stained for CD63 antigen. Volumetric control was used to estimate concentration of CD47^+^CD63^+^ EVs. One representative experiment is presented out of 2 performed.

NanoSight analysis of bulk EVs isolated from Jurkat T cells indicated a mean size of 122 ± 3 nm (SE) and a mode of 101.6 ± 3.7 nm (Figs 1a and S1). EVs released from the CD47-deficient Jurkat mutant JinB8 showed a similar size distribution but averaged larger than EVs from WT Jurkat cells (mean 140.1 ± 2.8 nm and mode 117.7 ± 6.8 nm, Fig. 1b).

### CD47 is present on a subset of CD63^+^ and MHC1^+^ EVs

Bulk EVs isolated from Jurkat T cell conditioned medium using a commercial polymeric precipitation method were fractionated using ~15 nm magnetic nanoparticles (MNPs) conjugated with antibodies specific for the exosome markers CD63, MHC1, and CD47. Efficiency of the capture using the CD63 antibody MNPs was estimated to be 83% for Jurkat T cell EVs and 88% for CD47-deficient JinB8 EVs based on recapture followed by flow analysis of Bodipy FL-labeled EVs from the two cell lines (Fig. S1).

Flow cytometry analysis using fluorescence thresholding with a PE-conjugated CD47 antibody was used to examine CD47 expression on the CD63^+^ and MHC1^+^ EV populations released by Jurkat cells (Fig. 1c). This analysis showed that 20.5% and 17.6%, respectively, of CD63^+^ and MHC1^+^ EVs are positive for CD47 (Fig. 1c). Analysis of CD63^+^ and MHC1^+^ EVs isolated from CD47-deficient JinB8 cells confirmed the specificity of detecting CD47 antibody binding to EVs (Fig. 1d).

Flow cytometry analysis using fluorescence thresholding was calibrated using 160–500 nm MegaMix-SSC fluorescent calibration beads to characterize the size distribution of CD63^+^ and MHC1^+^ EVs that express CD47 (Figs S2 and S3, Fig. 1e). Both subsets of CD47^+^ EVs are predominantly 160–240 nm in size, but larger CD47^+^/MHC1^+^ EVs (black bars) were more prevalent than CD47^+^/CD63^+^ EVs (red bars). Flow analysis of CD63 staining of EVs capture using anti-CD47 MNPs confirmed the presence of EVs bearing both surface proteins (Fig. 1f).

### RNA Sequencing Work Flow

Because transfer of RNAs is a major mediator of EV functional effects on target cells, we performed a global analysis of the small (<150 nt) noncoding RNA content in Jurkat T cell-derived CD63^+^, MHC1^+^, and CD47^+^ EVs. Total RNA was extracted from the captured CD63^+^, MHC1^+^, and CD47^+^ EVs and from the respective uncaptured fractions of CD63^−^, MHC1^−^, and CD47^−^ EVs using a Qiagen miRNeasy micro kit that preserves all classes of RNAs. The RNA quality was accessed by Bioanalyzer (Fig. S4). Libraries enriched in smaller RNAs were prepared using the Clontech SMARTer small RNA-seq kit for the Illumina NextSeq. 500 platform combined with bead-based size selection. Sequencing was done with 12.5 million reads per sample with 75 bp reads, multiplexing 32 samples on a 400 million read flow cell.

The small RNA sequencing data was analyzed using DNASTAR with three methods. For total RNA evaluation, the standard RNA sequencing work flow was used (Fig. S5 Method I). RNA sequencing reads were aligned using the DNASTAR ArrayStar workflow to either small coding/noncoding RNAs (CDS, rRNA, tRNA, ncRNA, tmRNA and miscRNA) or to miRNA (hairpin.fa and mature.fa) reference databases. To further evaluate small RNAs, we used miRNA sequencing DNASTAR - ArrayStar work flow, which evaluates long noncoding RNAs and other small RNAs (Fig. S5 Method II). To further evaluate miRNA, we used a SeqMan NGen assembly aligned with miRbase, and analysis was performed using the RNA-Seq analysis workflow of ArrayStar (Fig. S5 Method III). A full description of the work flows is in the Methods section.

### CD63^+^, CD47^+^ and MHC1^+^ EVs differentially enrich noncoding RNAs

**Figure 2:**
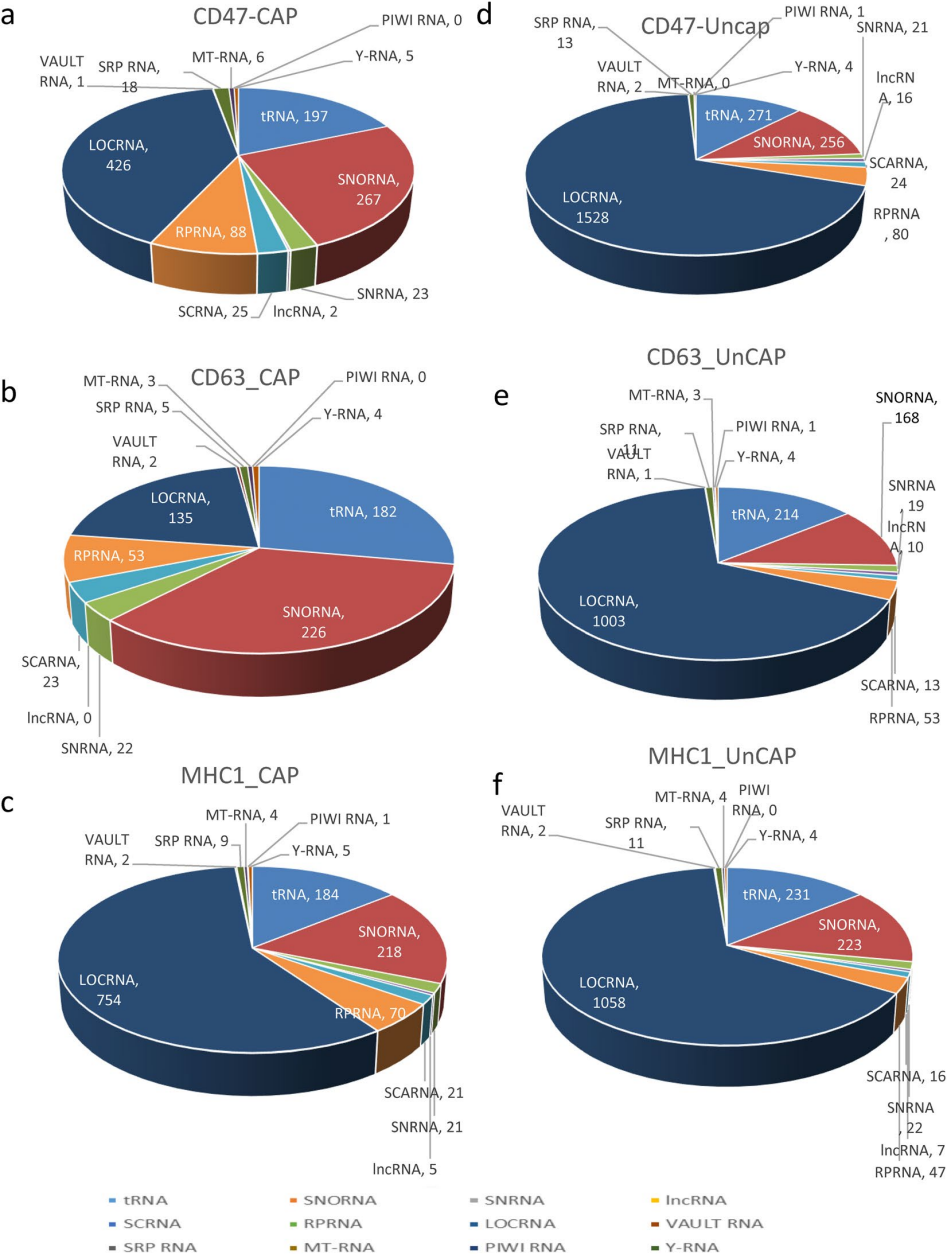
Noncoding RNA content of CD47^+^, CD63^+^ and MHC1^+^ captured EVs versus the respective uncaptured EVs. (**a–c**) Individual classes of noncoding RNAs were extracted from linear total RPKM Gene Table by name and a table was generated for each type of RNA. The cutoff threshold 0.02 log2 total RPKM was used to filter RNAs isolated from CD47^+^, CD63^+^ and MHC1^+^ EVs, and the numbers of mapped genes in each class are shown in pie charts. **(d–f)** Similarly, the number and type of non-coding RNAs identified in CD47^−^, CD63^−^ and MHC1^−^ EVs and are shown as pie charts. Abbreviations: PIWI RNA, piwi-interacting RNA; Y-RNA, small non-coding RNA components of the Ro ribonucleoprotein particle; MT-RNA, mitochondrial RNA; SRP RNA, signal recognition particle RNA; Vault RNA RNA family of the vault ribonucleoprotein complex; SCA RNA, small Cajal body-specific RNA; SnRNA, small nuclear RNA; lncRNA, long non-coding RNA; SnoRNA, small nucleolar RNAs; RPRNA, ribosomal protein RNA; tRNA, transfer RNA; LOCRNA, nonannotated gene transcripts.

Alignment of the RNA sequencing data using method (I) to align coding and non-coding RNAs to a reference genome revealed that all uncaptured EV fractions following depletion of EVs expressing each of the three markers have similar noncoding RNA compositions (Fig. 2d–f and Table A). EVs captured using anti-CD63 showed the greatest enrichment of other classes of noncoding RNAs relative to nonannotated RNAs (locRNA, Fig. 2b). EVs captured using anti-CD47 showed intermediate enrichment, whereas EVs captured using anti-MHC1 showed only modest depletion of locRNAs relative to the uncaptured controls (Fig. 2a,c). Comparing each type of antibody-captured EVs revealed that CD47^+^ and CD63^+^ EVs have similar overall noncoding RNA composition profiles except for a lower number of snRNAs in CD47^+^ EVs. On the other hand, MHC1^+^ EVs have a more distinct RNA profile compared to CD63^+^ or CD47^+^ EVs. MHC1^+^ EVs contain greater numbers of locRNAs and tRNAs (Fig. S6a–c). The statistical t-test analysis showed significant differences between the numbers of identified transcripts in the respective captured and uncaptured EVs: CD63^+^/CD63^−^ (120 transcripts with FDR < 0.1), MHC1^+^/MHC1^−^ EVs (49 transcripts with FDR < 0.1), and CD47^+^/CD47^−^ EVs (606 transcripts with FDR < 0.1,Table B).

### CD63^+^, CD47^+^ and MHC1^+^ EVs enrich different small non-coding RNAs

**Figure 3:**
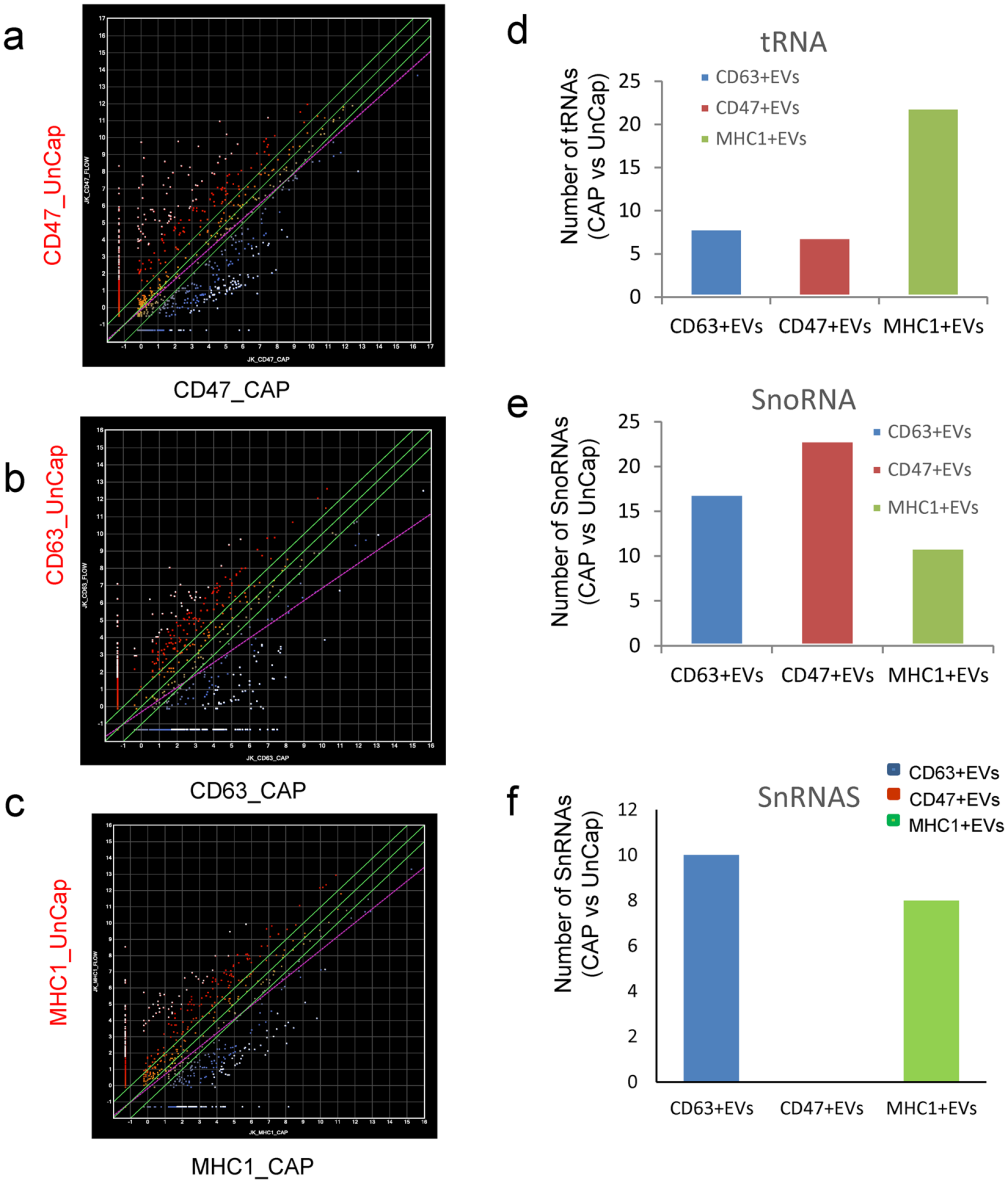
Enrichment of small RNAs in antibody captured EVs. **(a)** Scatter graph of data from 3 biological replicates comparing RNA abundance in CD47^+^ and CD47^−^ EVs. A total of 680 transcripts were significantly enriched or depleted in captured EVs (CD47_CAP) versus uncaptured EVs (CD47_UnCap, p < 0.05). Data are presented as log base 2 of total RPM. White dots indicate >8-fold enrichment or depletion. Red and blue indicate >2-fold enrichment or depletion, respectively. (**b)** Scatter graph of data from 2 biological replicates comparing RNA abundance in CD63^+^ and CD63^−^ EVs. A total of 1339 transcripts were significantly enriched or depleted in captured EVs (CD63_CAP) versus uncaptured EVs (CD63_UnCap)**. (c)** Scatter graphs of data from 2 biological replicates comparing RNA abundance in MHC1^+^ and MHC1^−^ EVs. A total of 782 were significantly enriched or depleted in captured EVs (MHC1_CAP) versus uncaptured EVs (MHC1_UnCap). **(d–e)** Graphs presenting the numbers of significantly enriched or depleted RNAs belonging to the indicated classes comparing captured versus uncaptured CD47^+^ CD63^+^ and MHC1^+^ EV samples.

Differences in the efficiency of packaging of specific RNAs into CD63^+^, MHC1^+^ and CD47^+^ EVs is one potential source for the observed heterogeneity in RNA contents between EVs expressing different surface markers. To assess the surface marker-dependence of RNA sorting into each subset of EVs we compared captured CD63^+^, MHC1^+^ and CD47^+^ EVs with uncaptured EVs lacking each respective marker and selected transcripts with at least 2-fold higher expression in captured versus uncaptured EVs and a minimum log_2_ total RPM = 2 (Fig. 3a–c). A total of 680 RNAs were significantly enriched or depleted ≥ 2-fold at p < 0.05 in captured versus uncaptured CD47^+^ EVs (Fig^.^ 3a). Comparing captured CD63^+^ versus uncaptured CD63^-^ EVs showed 1338 RNAs with ≥ 2-fold differential expression (Fig. 3b). Captured versus uncaptured MHC1^+^ EVs showed 799 ≥ 2-fold differentially expressed transcripts (Fig. 3c).

We next determined the number of transcripts in specific classes of RNAs that were significantly enriched in the three subsets of EVs. Similar numbers of lncRNAs and locRNAs were enriched by the three antibodies, whereas CD47^+^ EVs showed a notable absence of snRNA enrichment but enrichment of more snoRNAs (Fig. 3d–g). In contrast, MHC1^+^ EVs showed enrichment of more tRNAs than either CD63^+^ or CD47^+^ EVs (Fig. 3d).

**Figure 4:**
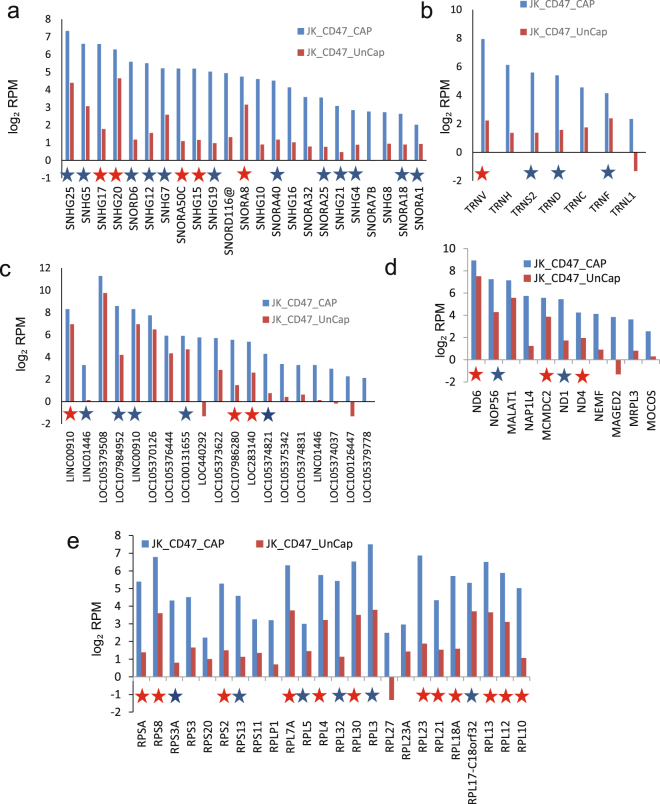
RNAs enriched in CD47^+^ EVs. Analysis of specific classes of RNAs in the 272 differentially expressed non-coding RNAs that were enriched ≥ 2-fold in CD47^+^ EVs versus CD47^−^ EVs from the scatter graph in Fig. 3a. A table was generated for each type of enriched RNA in CD47^+^ EV samples. **(a)** log_2_ total RPM values are plotted for the indicated snoRNAs from CD47^+^ EVs (blue bars) and uncaptured CD47^−^ EVs. **(b)** Log_2_ total RPM values are plotted for the indicated tRNAs from CD47^+^ EVs (blue bars) and uncaptured CD47^−^ EVs. **(c)** Log_2_ RPM values are plotted for the indicated lncRNAs from CD47^+^ EVs (blue bars) and uncaptured CD47^−^ EVs. **(d)** Log_2_ total RPM values are plotted for the indicated mitochondrial and other RNAs from CD47^+^ EVs (blue bars) and uncaptured CD47^−^ EVs. **(e)** Log_2_ total RPM values are plotted for the indicated ribosomal protein RNAs from CD47^+^ EVs (blue bars) and uncaptured CD47^−^ EVs. Blue stars indicate RNAs that are also enriched in CD63^+^ or MHC1^+^ EVs. Red stars indicate RNAs enriched in all three types of captured EVs.

Examination of specific RNAs that were enriched in each type of EVs revealed more heterogeneity. CD47^+^ EVs were enriched ≥ 2-fold in 272 transcripts, which included small nucleolar RNAs, lncRNAs, ribosomal protein RNAs, tRNAs and others (Fig. 4a–e and Supplemental Table 1, Fig. 3a). Among them, 205 with ≥ 8-fold enrichment (Fig. 3a) and 65 of those genes were statistically significant at p < 0.05, including long noncoding and nonannotated RNAs, tRNA, and ribosomal protein RNAs.

**Figure 5:**
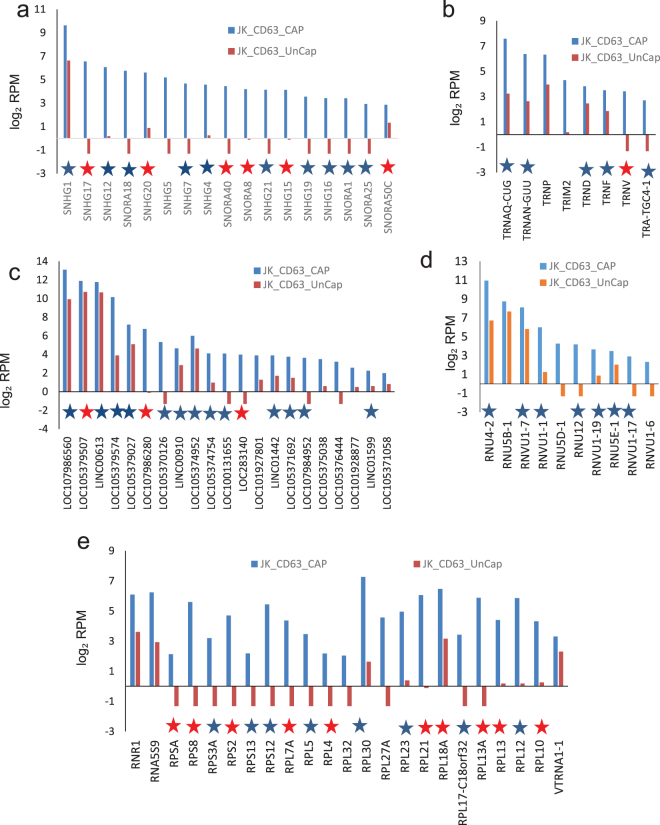
RNAs enriched in CD63^+^ EVs. Analysis of specific classes of RNAs in the 271 differentially expressed non-coding RNAs that were enriched ≥ 2-fold in CD63^+^ EVs versus CD63^-^ EVs were extracted from the scatter graph in Fig. 3b. A table was generated for each type of upregulated RNAs of captured CD63^+^ EVs. **(a)** Log_2_ total RPM values are plotted for the indicated snoRNAs from CD63^+^ EVs (blue bars) and uncaptured CD63^-^ EVs. **(b)** Log_2_ total RPM values are plotted for the indicated tRNAs from CD63^+^ EVs (blue bars) and uncaptured CD63^-^ EVs. **(c)** Log_2_ total RPM values are plotted for the indicated lncRNAs from CD63^+^ EVs (blue bars) and uncaptured CD63^-^ EVs. **(d)** Log_2_ total RPM values are plotted for the indicated small nuclear RNAs from CD63^+^ EVs (blue bars) and uncaptured CD63^-^ EVs. **(e)** Log_2_ total RPM values are plotted for the indicated ribosomal protein RNAs from CD63^+^ EVs (blue bars) and uncaptured CD63^-^ EVs. Blue stars indicate RNAs that are also enriched in CD47^+^ or MHC1^+^ EVs. Red stars indicate RNAs enriched in all three types of captured EVs.

CD63^+^ EVs were enriched in 271 transcripts, which included small nucleolar RNAs, lncRNAs, ribosomal protein RNAs, small nuclear RNAs and tRNAs (Fig. 5a–e and Supplemental Table 2, Fig. 3b). Among them, enrichment for 36 genes was statistically significant at p < 0.05. They included SnoRNAs, nonannotated RNAs, ribosomal protein RNAs, and a minor population of long noncoding RNAs.

**Figure 6:**
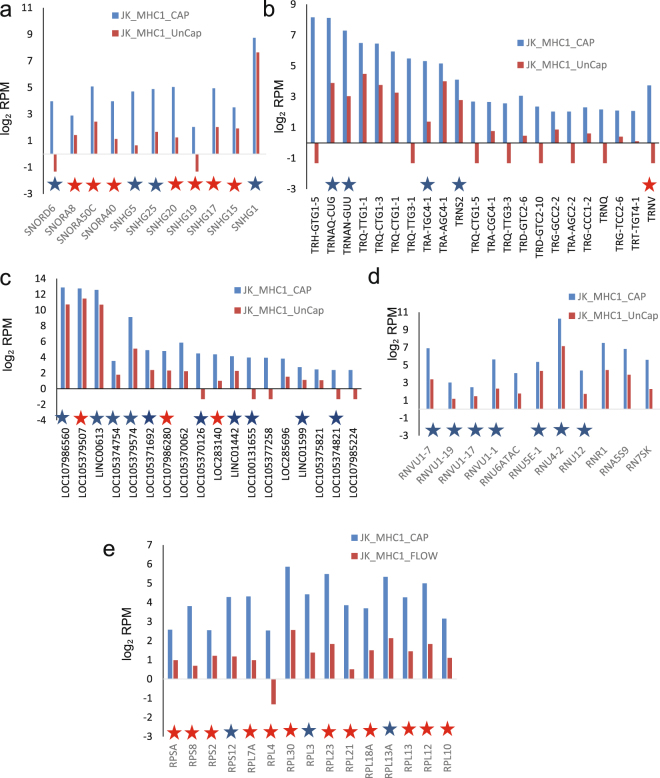
RNAs enriched in MHC1^+^ EVs. Analysis of specific classes of RNAs in the 268 differentially expressed non-coding RNAs that were enriched ≥ 2-fold in MHC1^+^ EVs versus MHC1^−^ EVs were extracted from the scatter graph in Fig. 3c. A table was generated for each type of upregulated RNAs of captured MHC1^+^ EVs. **(a)** Log_2_ total RPM values are plotted for the indicated snoRNAs from MHC1^+^ EVs (blue bars) and uncaptured MHC1^−^ EVs. **(b)** Log_2_ total RPM values are plotted for the indicated tRNAs from MHC1^+^ EVs (blue bars) and uncaptured MHC1^−^ EVs. **(c)** Log_2_ total RPM values are plotted for the indicated lncRNAs from MHC1^+^ EVs (blue bars) and uncaptured MHC1^−^ EVs. **(d)** Log_2_ total RPM values are plotted for the indicated small nuclear RNAs from MHC1^+^ EVs (blue bars) and uncaptured MHC1^−^ EVs. **(e)** Log_2_ total RPM values are plotted for the indicated ribosomal protein RNAs from MHC1^+^ EVs (blue bars) and uncaptured MHC1^−^ EVs. Blue stars indicate RNAs that are also enriched in CD63^+^ or MHC1^+^ EVs. Red stars indicate RNAs enriched in all three types of captured EVs.

MHC1^+^ EVs were enriched in 265 transcripts, which included tRNAs, lncRNA, ribosomal protein RNA, small nuclear and nucleolar RNAs (Fig. 6a–e and Supplemental Table 3, Fig. 3c). MHC1^+^ EVs contained the largest number of enriched tRNAs, but had the least number of enriched lncRNAs, ribosomal protein RNAs, and small nucleolar RNAs. Of these only 4 were significant at p < 0.05.

Further comparison of transcript levels between CD47^+^ and CD63^+^ EVs, identified 628 (≥2-fold) differentially expressed RNAs (Fig. S6a), and 25 of these were significant at p < 0.05 (Fig. S6b), while 689 (≥2-fold) were differential enriched between CD47^+^ and MHC1^+^EVs (Fig. S6c), and 42 transcripts were significant at p < 0.05 (Fig. S6d). A total of 483 RNAs were differential expressed (≥2-fold) between CD63^+^ and MHC1^+^ EVs (Fig. S6e), but differences in only 9 transcripts were significant p < 0.05 (Fig. S6f). Therefore, EVs with CD47, CD63 and MHC1 surface markers have similar overall contents of non-coding RNAs, but each subset selectively enriches specific classes of non-coding RNAs.

Enrichment of RNAs encoding ribosomal proteins was conserved among CD63^+^, MHC1^+^ and CD47^+^ EVs (Figs 4e, 5e and 6e). Twelve of the 15 ribosomal protein RNAs enriched ≥ 2-fold in MHC1^+^ EVs were also enriched in CD47^+^ and CD63^+^ EVs, and the remaining 3 were enriched ≥ 2-fold in two of the three subsets. Six snoRNAs (SNHG17, SNHG20, SNORA50C, SNHG15, SNORA8, and SNORA40) were enriched in all three types of captured EVs, and most others were enriched in 2 of the 3 (Figs 4a, 5a and 6a). Long noncoding RNA enrichment showed more heterogeneity, with only LOC105379507, LOC107986280, and LOC283140 consistently enriched in EVs expressing each of the three markers (Figs 4c, 5c and 6c). Among the tRNAs, only TNRV was ≥ 2-fold enriched in EVs expressing each of the three markers (Figs 4b, 5b and 6b).

### CD63^+^ and CD47^+^ EVs contain more miRNAs as compared to MHC1^+^ EVs

**Figure 7:**
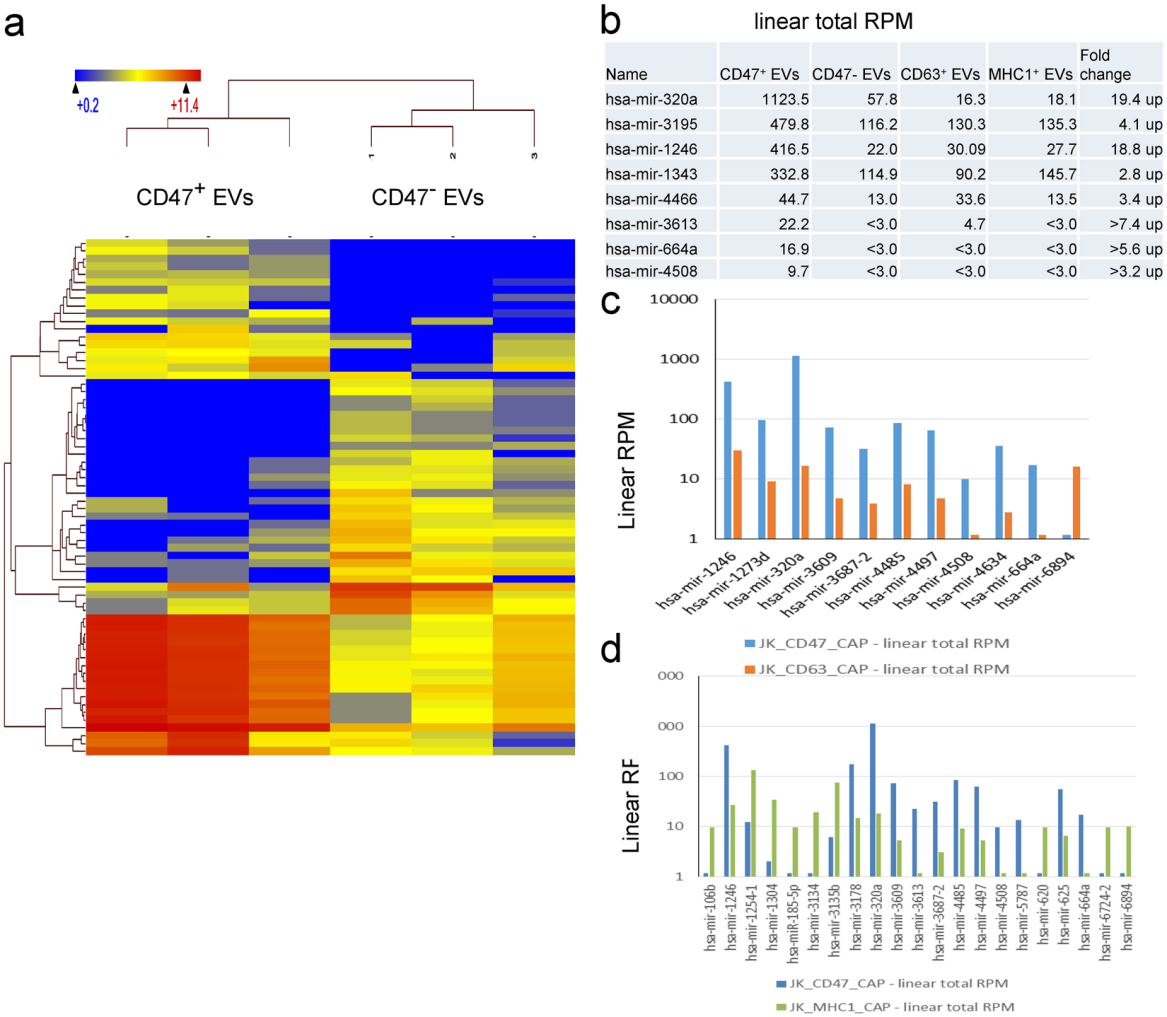
Micro-RNA enrichment in captured EVs determined by RNAseq. **(a)** Heat map of hierarchical clustering of 109 miRNAs (All species) differentially enriched in CD47^+^ versus CD47^−^ EVs at p < 0.05. **(b)** 27 miRNAs were upregulated, and 5 hsa-miRNA are enriched only in CD47^+^ EVs. **(c)** A comparison of CD47^+^ and CD63^+^ EVs shows differential expression of 100 miRNAs (All species) at p < 0.05 using log base 2 of total RPM, (CD47_CAP and CD63_CAP with Student’s t-test), and 11 hsa-miRNAs are presented in the graph. **(d)** A comparison of CD47^+^ and MHC1^+^ EVs shows differential expression of 112 miRNAs (All species) at p < 0.05 using log base 2 of total RPM (CD47_CAP and YCD63_CAP with Student’s-test), and 21 hsa- miRNA are presented in the graph.

To align miRNAs in the RNAseq data, fastq files were assembled using SeqMan with NGen miRbase 2.1 as the reference. The percentages of aligned sequences are shown in the bar graph in Fig. S7a. A scatter plot of CD47^+^ vs CD47^−^ miRNA expression values (RPM) determined by RNA sequencing analysis using a statistical t-test identified a total of 186 miRNAs with p < 0.05 (all microRNA species included, Figs 7a and S7c). Among them 8 human miRNAs were enriched in CD47^+^ EVs (Fig. 7b). Eleven human miRNAs are differentially regulated in CD47^+^ EVs versus CD63^+^ EVs (Fig. 7c), while 21 human miRNAs are found differentially expressed between CD47^+^ EVs and MHC1^+^ EVs (Fig. 7d).

**Figure 8:**
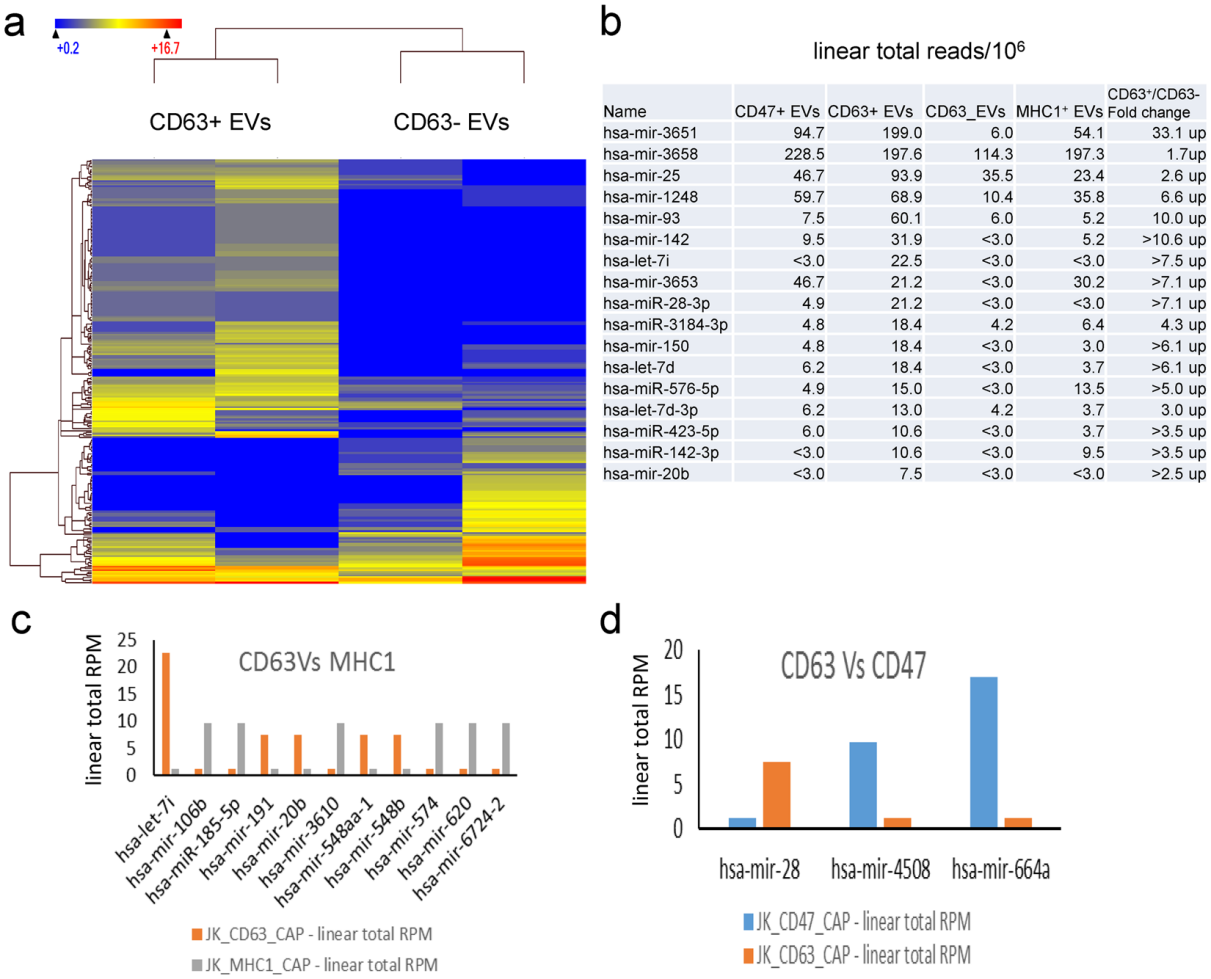
Differential enrichment of miRNAs in CD47^+^, CD63^+^ and MHC1^+^ EVs. **(a)** Hierarchical clustering of 208 miRNAs (p < 0.05 Student-t test, All species) differentially enriched in CD63^+^ versus CD63^-^ EVs. **(b)** Out of 208 miRNAs (39 hsa-miRNAs), 11 hsa-miRNA were differentially expressed in CD63^+^ EVs. **(c)** A comparison of CD63^+^ and MHC1^+^ EVs shows differential expression of 11 hsa-miRNAs. **(d)** A comparison of CD63^+^ and CD47^+^ EVs shows differential expression of 3 hsa-miRNAs.

The t-test for CD63 captured versus uncaptured EVs showed that a total of 208 miRNAs were differentially expressed at p < 0.05 (All species included, Figs 8a and S7c). Among them miR-3539-3p, miR-93-5p, miR-142, miR-150, miR-28-3p, miR-423-5p, let-7d and let-7i are highly enriched in CD63^+^ EVs (Red font-Fig. 6b). Some of the same enriched miRNAs were also identified in CD47^+^ and MHC1^+^ EVs (Blue font). Conversely, eleven human miRNAs are differentially enriched in CD63^+^ EVs versus MHC1^+^ EVs (Fig. 8c), while only 3 miRNAs are differentially enriched between CD63^+^ EVs versus CD47^+^ EVs (Fig. 8d).

A scatter plot of miRNAs (all species) between MHC1^+^ versus MHC1^−^ EVs showed that 135 miRNAs are differentially regulated at p < 0.05 (Fig. S7d). Among them miRNA-106b, miR-185-5p, miR-3610, miR-620 and miR-663 were enriched with MHC1^+^ EVs (Fig. S8a) but their linear RPM indicate that MHC1^+^ EVs contain substantially less miRNA than CD63^+^ and CD47^+^ EVs. Further comparison between MHC1^+^ and CD47^+^ EVs show only 5 hsa-miRNA are differentially expressed (Fig. S8b). However, interpretation of this comparison is limited by the low RPM for this RNA class in MHC1^+^ EVs.

### miRNA microarray analysis of CD63^+^ and CD47^+^ EVs captured using streptavidin magnetic beads

**Figure 9:**
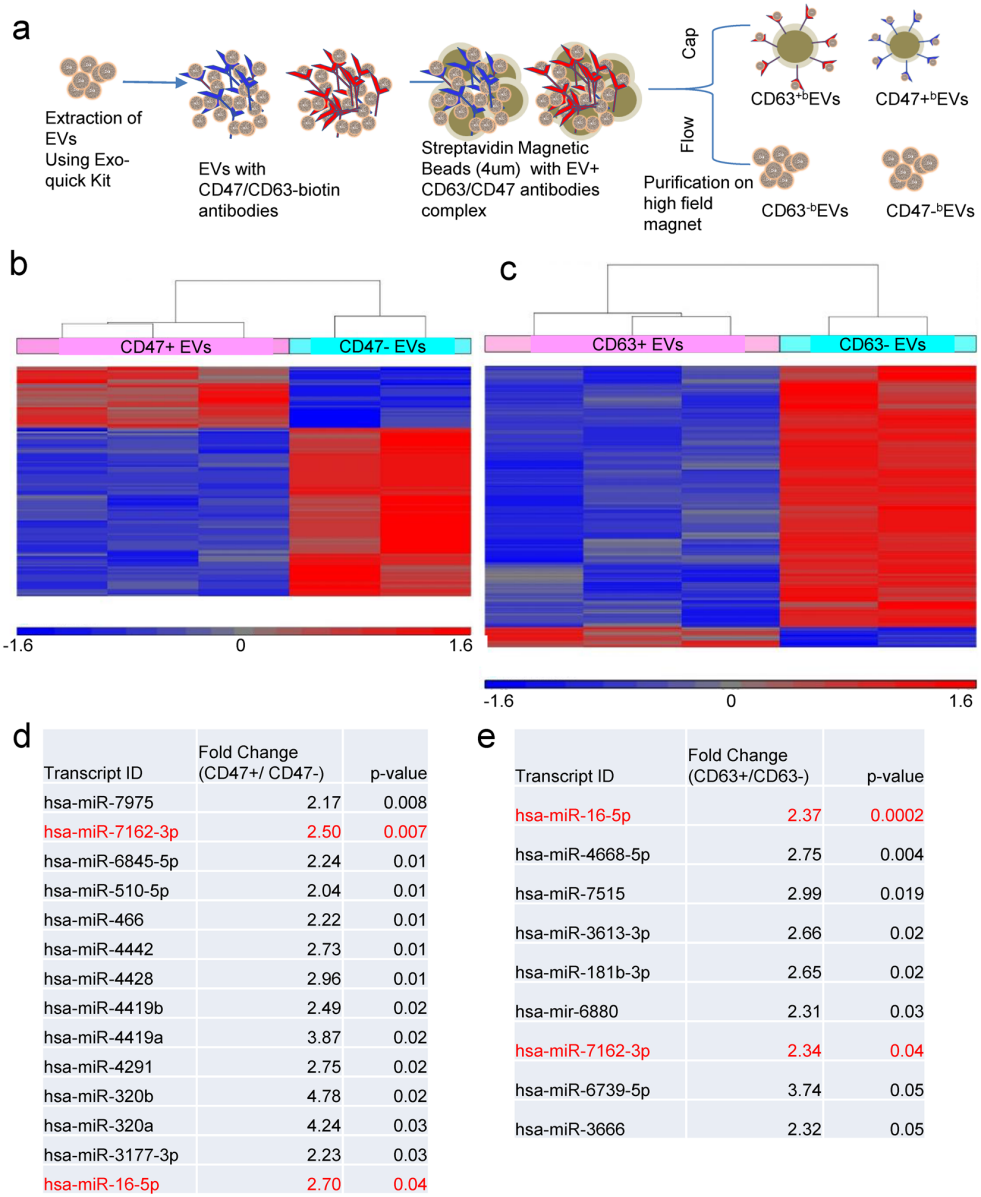
Micro-RNA enrichment in captured EVs determined by microarray analysis. **(a)** Preparation of CD47^+^ and CD63^+^ EVs for miRNA microarray analysis. **(b)** Hierarchical clustering of human miRNAs identifies differential expression between CD47^+b^ and CD47^−b^ EVs (Student-t test). **(c)** Heat map of hierarchical clustering Human miRNA differentially expressed between CD63^+b^/CD63^−b^ EVs. **(d)** The table presents enriched hsa-miRNAs in CD47^+ ^EVs. **(e)** The table presents enriched hsa-miRNAs in CD63^+^ EVs.

To independently confirm and extend the above RNAseq analysis of miRNAs, bulk Jurkat T cell-derived EVs were captured using biotinylated CD63 or CD47 antibodies coupled to streptavidin magnetic beads (Fig. 9a). The CD63 and CD47 captured and uncaptured fractions of EVs were subjected to the same total RNA extraction as above and analyzed using miRNA microarrays. The miRNA microarray results show differentially expressed miRNAs in the captured versus uncaptured EVs (Fig. 9b,c). The miRNAs highly enriched in the respective captured versus uncaptured EVs are shown in Fig. 9d,e. The microarray data confirmed some of the RNAseq data, including the enrichment of miR-320 in CD47^+^ EVs. Further comparison of the miRNA microarray data for CD47^+^ and CD63^+^ EVs isolated by magnetic bead capture with RNA sequencing data for the corresponding EVs isolated by MNP capture confirmed significant enrichment of 21 miRNAs and significant depletion of 15 miRNAs. However, 24 other miRNAs showed divergent changes using the two methods (Fig. S8c).

### Validation of small non-coding RNA-Sequencing Analysis

**Figure 10:**
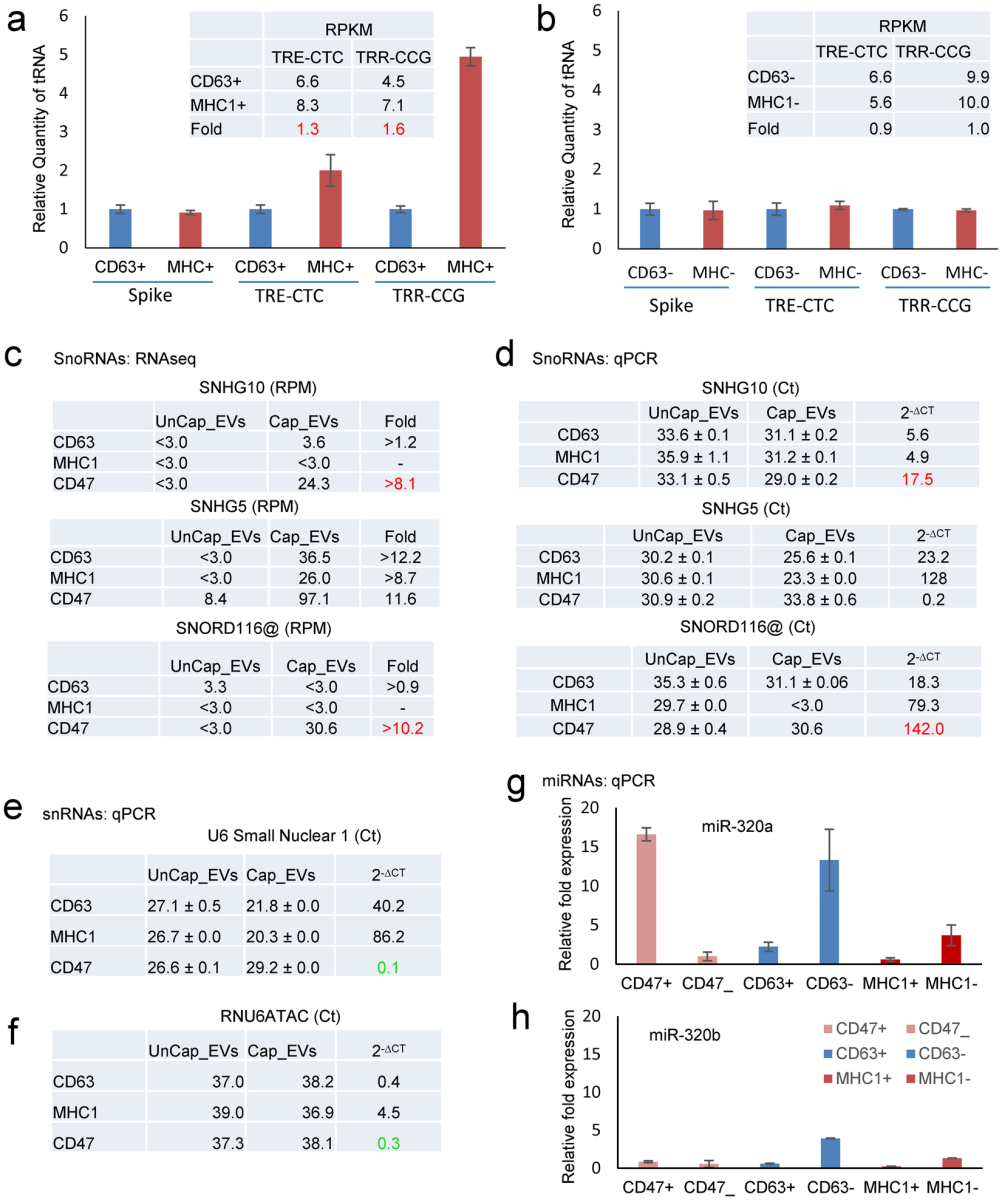
Validation of small RNA enrichment in CD47^+^, CD63^+^ and MHC1^+^ captured EVs. (**a,b**) Validation of tRNAs using rtStar™ Pre-designed Human tRNA primer sets for TRE-CTC, TRR-CCG and internal spike control using total RNA from captured and uncaptured CD63 and MHC1 EVs. (**c**) The table presents RPM of SnoRNAs in CD47^+^, CD63^+^, and MHC1^+^ EVs. (**d**) Expression of SNHG5, SNHG10 and SNORDA116@ snoRNAs using total RNA from captured and uncaptured CD47, CD63 and MHC1 EVs via TaqMan real-time PCR. (e) U6 using total RNA from captured and uncaptured CD47, CD63 and MHC1 EVs via real-time PCR. (**f**) snRNA expression of RNU6ATAC using total RNA from captured and uncaptured CD47, CD63 and MHC1 EVs via TaqMan assay. (**g,h**) miRNA expression of mir-320a and mir-320b using total RNA from captured and uncaptured CD47, CD63 and MHC1 EVs determined using real-time PCR.

Our noncoding RNAseq analysis using Method II indicated that MHC^+^ EVs contain higher levels of some tRNAs than CD63^+^ EVs, including TRR-CCG (Fig. 10a). To confirm this finding we performed tRNA real-time PCR analysis for CD63^+^ and MHC1^+^ EVs and the corresponding depleted EV fractions (Fig. 10b and c). As expected, CD63^−^ and MHC1^−^ EVs had similar levels of these tRNAs, whereas MHC1^+^ EVs had higher levels of TRE-CTC and TRE-TRR compared to CD63^+^ EVs.

qPCR was also used to validate selected differentially enriched small nucleolar RNAs identified using RNAseq. The former data indicated that SNHG5 is enriched in CD63^+^, CD47^+^, and MHC1^+^ EVs (Figs 4a, 5a, 6a and 10c). Analysis of SNHG5 using a TaqMan assay confirmed its enrichment in CD63^+^, and MHC1^+^ EVs (Fig. 10d). The RNAseq data indicated that SNHG10 and SNORD116@ are highly enriched only in CD47^+ ^EVs (Fig. 10c), and TaqMan real-time PCR data confirmed this finding (Fig. 10d).

CD47^+ ^EVs had fewer snRNAs as compared to CD63^+^ EVs and MHC1^+^ EVs (Fig. 3f), and we confirmed decreased expression of U6 Small Nuclear 1 using real time PCR analysis (Fig. 10e). The snRNA profile of MHC1^+^ EVs showed high expression of RNU6ATAC (Fig. 6f), and we have confirmed this using a TaqMan assay (Fig. 10e).

miRNA sequencing analysis (Method III) revealed high expression of miRNA-320a in CD47^+^ EVs. This was validated using magnetic bead captured CD47^+^ and CD63^+^EVs (Fig. 9a) with miRNA microarray analysis (Fig. 9b–e). Both methods showed that CD47^+^ EVs are enriched with miRNA-320a (Figs 7b and 9d). Microarray analysis also showed high expression of miRNA-320b. We further analyzed these microRNAs using real time PCR analysis and confirmed that miRNA-320a but not miRNA-320b is enriched in CD47^+^ EVs but not in CD63^+^ or MHC1^+^ EVs (Fig. 10g and h).

## Discussion

Our previous analysis of EVs released by Jurkat T cells that express CD47 with EVs released by a CD47-deficient Jurkat mutant identified differences in their content of coding mRNAs, but it was unclear whether CD47 played a direct or an indirect role in determining which mRNAs are packaged into EVs^22^. Here we show that the small noncoding RNA content of CD47^+^ EVs differs from that of CD47^−^ EVs released by the same cells. Analysis of EVs expressing or lacking the surface markers CD63 and MHC1 revealed that EVs expressing different surface markers share some transcripts in common, but each subset of EVs also shows distinct enriched transcripts relative to EVs expressing different markers and relative to EVs lacking the respective markers. CD47^+^, CD63^+^ and MHC1^+^ EVs are enriched in distinct subsets of noncoding RNAs. CD63^+^ EVs have a higher number of enriched miRNAs, but most are shared with CD47^+^ and MHC1^+^ EVs. However, CD47^+^ EVs enrich specific miRNAs that are not enriched in CD63^+^ or MHC1^+^ EVs.

The present data provides a comprehensive analysis of multiple classes of smaller noncoding RNAs. CD47, CD63 and MHC1 antibodies each capture a minority of EVs, and some EVs express more than one of these markers. However, CD47-, CD63- and MHC1-depleted fractions retain the majority of the total EV-associated RNA content. Based on the high efficiency of recapture using MNPs with the same antibody, this is not an artifact of the capture method, but instead probably indicates that a substantial fraction of EVs released by Jurkat T cells lack sufficient copies of the three proteins to enable antibody capture. The substantial differences in the degree of enrichment for specific RNAs in CD47^+^, CD63^+^ and MHC1^+^ EVs suggests heterogeneity in the targeting of different classes of RNAs and specific transcripts and that these three surface protein markers identify functionally distinct classes of EVs. We can only draw conclusions for the single cell type used, but such functional heterogeneity probably exists in EVs released by other cell types.

Our results show that CD47^+^, CD63^+^ and MHC1^+^ EVs predominantly contain long-coding RNAs, tRNAs, small nucleolar RNAs, ribosomal protein RNAs, and a small fraction of miRNAs. CD63^+^ and CD47^+^ EVs show more overlap with each other in their miRNA content, while MHC1^+^ EVs are more distinct. MHC1^+^ EVs also concentrate a greater number of tRNAs than do CD47^+^ and CD63^+^ EVs. In contrast CD63^+^ and MHC1^+^ EVs concentrate similar populations of small nuclear RNAs, whereas CD47^+^ EVs do not significantly enrich any small nuclear RNAs. Previous studies have identified mechanisms that enable sequence-specific sorting of miRNAs into EVs, but the present results suggest that other classes of noncoding RNAs are also sorted into EVs, and different sorting mechanisms must exist to target different RNAs to EVs identified by expression of each of these three surface proteins.

Further, we have shown that purification by magnetic capture of EVs expressing CD63 and CD47 using antibody-modified MNPs or streptavidin-magnetic beads results in similar miRNA profiles despite differences related to chip content, sequence mapping algorithms and the mirBase dataset content. Data from the miRNA microarrays generally confirmed the differences identified using miRNAseq analysis, but the microarrays have higher sensitivity for detecting miRNA given the low total percentage of miRNAs in the preparations used for generating the sequencing libraries. CD63^+^ EVs captured using MNPs were enriched in 15 unique miRNAs, and 10 unique miRNAs were enriched in CD47 and MHC1 captured EVs. Microarray analysis showed that the CD47^+^ EVs captured with beads yield more miRNA than CD63^+^ EVs.

Many of the miRNAs identified are unique to EVs bearing one of the three surface markers examined and are enriched in these EVs relative to the respective uncaptured EV fractions. Further studies are required to determine the molecular mechanisms by which miRNAs are selectively targeted to CD47^+^, CD63^+^ and MHC1^+^ EVs. It is unclear whether the surface proteins play a direct or indirect role in the loading of specific RNA contents into EVs. miRNA sorting into EVs can be regulated by chromosome location^23^, regulation of miRNA biosynthesis^24^, Ras activation^25^, AGO2 activity^26^, neural sphingomyelinase 2 activity^27^, and sequence-specific sorting that may be mediated by several RNA-binding proteins^13,28,29^. Sequence-specific sorting is an attractive mechanism to account for the marker-dependent differences reported here, but further study is needed to define candidate motifs unique to EVs expressing each surface marker and identify RNA binding proteins that may recognize these motifs.

Another important conclusion is that none of these three proteins is a universal marker for EVs. A major fraction of EVs and their noncoding RNA content were not captured by any of the three antibodies used in this study. The copy numbers of these surface proteins may be a limiting factor. A previous study determined that 141,000 copies/cell of a surface protein resulted in 30–70 copies/EV^30^. The reported density of CD47 on a typical cell is lower (~250 molecules/µm^2,^^31^, which corresponds to ~25 copies/120 nm EV. The minimal copy number for immunocapture by MNPs or beads remains to be determined. Because CD47 also interacts laterally with other membrane proteins and undergoes clustering, it is also possible that CD47 may be excluded from a fraction of the EVs released by Jurkat T cells. Apart from their heterogeneity as surface markers, a more important question concerns the potential roles CD47, CD63, and MHC1 play in determining the functional heterogeneity of EVs.

miRNAs that are specifically enriched in CD47^+^ EVs could have translational implications. CD47^+^ EVs are enriched in miR-320a, which has been associated with cardiac ischemia/perfusion injury by targeting heat shock protein-20^32^. CD47 is a major regulator of cell and tissue responses to stress^33^, and CD47 null mice or wild type mice following CD47 blockade are protected from cardiac, hepatic, vascular, and renal ischemic injuries^34–38^. Regulation of EV miRNA composition is one potential mechanism by which therapeutic blockade of CD47 confers tissue protection in treated animals. Future studies will also explore whether disease-associated miRNAs are enriched in EVs that bear specific surface markers. If so, immunoaffinity purification could enable more sensitive less invasive diagnostic procedures utilizing diseased patient sera or other body fluids. The small nucleolar RNAs SNHG10 and SNORD116@, which are enriched selectively in CD47^+^ EVs, were identified as a prognostic marker in human lung adenocarcinomas^39^ and a marker for progression of gastric cancer, respectively^40^. In this context, the elevated expression of CD47 in many cancers could result in enrichment of tumor-associated miRNAs and other noncoding RNAs in CD47^+^ EVs^33^. Therefore, immunoaffinity enrichment of CD47^+^ EVs from blood and other body fluids could increase the sensitivity for detecting cancer-associated noncoding RNAs in liquid biopsies.

## Materials and Methods

### Materials

The Jurkat T cell line (E6.1) was purchased from ATCC, and the CD47-deficient Jurkat mutant JinB8 were provided by Dr. Eric Brown, Genentech^41^. The cells were maintained using RPMI 1640 containing 10% FBS, glutamine, penicillin, and streptomycin (Gibco). The Jurkat and JinB8 T cells used for experiments were maintained less than 6 weeks in culture.

### EVs Isolation and labeling

The cultured Jurkat and JinB8 T cells were washed with PBS followed by a second wash with HITES medium (DMEM/F12 supplemented with, bovine serum albumin, hydrocortisone, insulin, transferrin, and trace elements)^42^. EVs were isolated from Jurkat T cells and were cultured overnight using HITES medium. The conditioned media from Jurkat T cells were collected, and cell debris were removed using centrifugation at 300 g for 5 minutes and 2500 g for 10 minutes respectively. Centrifuged media were concentrated using Ultra-4 centrifugal filters to about 1 ml volume and further centrifuged at 10,000 g for 10 minutes. EVs were isolated using an Exo-Quick kit (SBI) by incubating overnight at 4 °C. Isolated EVs were labeled with Bodipy-FL according to the manufacturer’s protocol (Millipore Sigma).

### Immunocapture of EVs

To characterize surface antigens on extracellular vesicles released by Jurkat cells, EVs were captured by CD63-MNPs, MHC I-MNPs or by CD47-MNPs as previously described^43^. Briefly, 15 nm magnetic nanoparticles (MNPs) (Ocean NanoTech, Springdale, AR) were activated and coupled with 1 mg of monoclonal antibodies anti-CD63 (Biolegend, San Diego, CA), anti-MHC (HLA-A,B,C Biolegend, San Diego, CA) or anti-CD47 clone B6H12 (eBiosciences) according to the manufacturer’s protocol. After coupling, antibody(Ab)-MNP complexes were suspended in 2 ml of wash/storage buffer and stored at 4 °C. To capture and stain surface antigens of EVs, MNP-Ab complexes (~3.9 × 10^12^ particles in 60 μl) were incubated with preparation of labeled EVs for 1 h at 4 °C, followed by addition of fluorescent monoclonal antibodies (anti–CD47-PE (Biolegend, San Diego CA for CD63-MNPs and MHC-MNPs capture or anti-CD63 PE (Biolegend, San Diego CA) for CD47-MNPs capture) and incubated for addition 20 minutes at room temperature. Controls were incubated with isotype control antibodies mouse IgG1k-PE (Biolegend, San Diego CA. Immunocaptured EVs stained for surface marker were separated from non-captured EVs and free floating antibodies on magnetic columns in a strong magnetic field generated by an OctoMacs magnet (Miltenyi Biotech), washed three times with 600 µl of washing buffer (0.5% bovine serum albumin, 2 mM EDTA in PBS). To elute captured EVs from columns, columns were removed from the magnet, flashed with 400 μl of PBS and fixed with 1.5% paraformaldehyde. The eluted complexes were analyzed on the LSRII cytometer triggering on fluorescence. Prior to analysis on the flow cytometer, a known number of 123-count eBeads (eBioscience, San Diego CA) counting beads were added to each sample. The volume of sample analyzed was estimated based on the number of 123 count eBeads acquired in each sample. Based on this volumetric measurement, the numbers of recorded events were normalized to calculate EV concentrations.

### Analysis of EVs with flow cytometry

Purified complexes were analyzed on an LSRII (BD Biosciences) flow cytometer with an inline 40 nm (Meissner, Camarillo, CA) filter equipped with 355, 407, 488, 532 and 633 nm lasers. The background level of fluorescence was evaluated with 0.1 μm filtered PBS, and the threshold was set to the lowest fluorescence channel that did not generate signal with this solution. Megamix-Plus SSC fluorescent calibration beads (Biocytex, Marseille, France) were used to estimate EV size. Data were acquired with Diva 6.3 and were analyzed with FlowJo software v9.4.9 (Treestar Software, Ashburn, OR).

### NanoSight Analysis

EV concentration and size measurements were performed on a NanoSight NS 300 (Salisbury,United Kingdom) equipped with a 405 nm laser and analyzed with NTA 3.0 (NanoSight NS300, Malvern Instruments, UK). The measurements were performed with constant sample flow using a syringe pump for 180 sec at camera level 13.

### RNA extraction, cDNA library and RNA sequencing

Jurkat T cell-derived EVs were isolated using the Exo-Quick kit, and CD63^+^, MHC1^+^, and CD47^+^ EVs were captured using MNPs conjugated with the respective antibodies. Total RNA was extracted from the captured CD63^+^, MHC1^+^, and CD47^+^ EVs and from the respective uncaptured fractions of CD63^−^, MHC1^−^, and CD47^−^ EVs with the Qiagen miRNeazy Mini kit (Cat# 217004) that preserves all kinds of RNAs. The RNA quality was assessed by the Bioanalyzer (Fig. S3). We used the Clontech SMARTer small RNA-seq kit (cat# 635029) for Illumina NextSeq. 500 platform with 17 cycles PCR. Purified cDNA libraries were quantified by Qubit 2.0 (Thermo Fisher Scientific Cat. # Q32866) and their quality assessed by the Agilent Bioanalyzer High Sensitivity DNA Kit (Agilent, Cat. # 5067-4626). Small RNA sequencing libraries were size selected (<150 bp) using the Agencourt AMPure beads (Beckman Coulter, Cat. No. A63880). KAPA qPCR Library Quantification Kit (KAPA Biosystems, Cat, # KK4824) was applied prior to pooling for the final libraries validation and normalization. The sequencing was done with 12.5 million reads per sample with 75 bp reads. (Multiplexing 32 samples on a 400 million read flow cell).

### RNA assembly and small RNA Sequencing Work Flow Analysis

The fast Q Illumina files were imported using QSeq/ArrayStar software package (DNASTAR, Inc.) Total RNA sequencing analysis was performed using default settings of QSeq/ArrayStar software by comparing the individual sequence reads to a whole genome reference using Homo sapiens. GRCh38 (Fig. S5(I). RNA sequencing reads were aligned by DNASTAR SeqMan NGen to either small coding/noncoding RNAs (CDS, rRNA, tRNA, ncRNA, tmRNA and miscRNA) or to miRNA reference (mature and precursors). We used miRNA sequencing DNASTAR ArrayStar work flow which databases. To yield long noncoding RNAs and other small RNAs (Fig. S5(II). To evaluate miRNA, we used a SeqMan aligned assembly with miRbase, and analysis was performed using RNA-Seq analysis (Fig. S5(III). The raw data is deposited in NCBI Gene Expression Omnibus (GEO): GSE103493.

### RNA Sequencing Analysis

Whole human genome Human reference was downloaded using Domain menu (EUKAROTA) and from the organism menu, *Homo sapiens* was selected. The fast Q Illumina files were imported using QSeq/ArrayStar software package (DNASTAR, Inc.) Total RNA sequencing analysis was performed using default setting of QSeq/ArrayStar software by comparing the individual sequence reads to a whole genome reference. The mapped and unmapped reads were shown in a Table S3. The normalization method RPKM used. Individual classes of noncoding RNAs were extracted from linear total RPKM Gene Table by name and a table was generated for each type of RNA. The cutoff threshold 0.02 log_2_ total RPKM was used to filter RNAs from CD47^+^, CD63^+^ and MHC1^+^ EVs, and the counts are shown in the pie charts (Fig. 2). Similarly, the number and type of non-coding RNAs identified in CD47^−^, CD63^-^ and MHC1^−^ EVs and are shown as pie charts. For defaults, Statistical setting of student’s t-test with a 1% Benjamini Hochberg False Discovery Rate (FDR) to compare captured and uncaptured treatments was used^44^.

### Small RNA Sequencing Analysis

A whole human genome reference was downloaded using the Domain menu (EUKAROTA) selecting *Homo sapiens* from the organism menu. The fastq Illumina files were imported using the QSeq/ArrayStar software package (DNASTAR, Inc.). The mapped and unmapped reads were shown in a Table S4. miRNA sequencing analysis was performed using QSeq Advanced Options Peak Detection setting, ‘Simple Peak Finder’ with minimum number of reads within the region set to 3 by comparing the individual sequence reads to a whole genome reference with features (CDS, rRNA, tRNA, ncRNA, tmRNA and miscRNA). The only normalization method available for this approach is RPM. Statistical analysis used Student’s t-test to compare captured and uncaptured treatments.

### miRNA- RNA Sequencing Analysis for miRNAs

An alternative analysis of miRNA expression used miRbase (hairpin.fa and mature.fa) as the reference in the RNA-Seq workflow of SeqMan NGen however, miRbase is still evolving^45^ and may have provisional annotations. Analysis of changes in miRNA expression was determined with the QSeq/ArrayStar software package (DNASTAR, Inc.) using RPM normalization in separate analyses. Statistical significance was calculated using a Student’s t-test to compare captured and uncaptured treatments.

### MiRNA Microarray analysis using CD63^+b^EVs and CD47^+b^EVs streptavidin magnetic bead pull down Assay

Jurkat T cell derived EVs were extracted using Exo-Quick kit as described above. The precipitated EVs were pre-incubated with CD63 and CD47-Biotin (Biolegend) for 30 min using a rocker at 4 °C. Then 100 µl of streptavidin beads washed with IX PBS were added to the EVs for 1 h. The bound (CD63 and CD47 captured) and unbound EVs (CD63 and CD47 uncaptured) were separated using a magnetic field, and harvested captured EVs were gently washed one time with PBS (Fig S9a). RNA was extracted from the captured CD63^+^, and CD47^+^ EVs and from the respective uncaptured fractions of CD63^-^, and CD47^−^ EVs in triplicate (n = 3) with the Qiagen miRNeazy Mini kit (Cat# 217004). The miRNA-microarray were performed as described previously^46^.The raw data of miRNA-microarray is deposited in NCBI Gene Expression Omnibus (GEO): GSE103310.

### t-RNA and miRNA Real-Time PCR

Total RNA was extracted from CD63^+^, MHC1^+^, and CD47^+^ EVs and from uncaptured fractions of CD63^-^, MHC1^−^, and CD47^−^ EVs with the Qiagen miRNeazy Mini kit. 100 ng of total RNA was used for the rtStar™ First-Strand cDNA Synthesis Kit, and real time PCR was performed using rtStar™ pre-designed Human tRNA Primer Sets for TRE-CTC, TRR-CCG along with Spike RNA as an internal control according to the manufacturer’s instructions. For miRNA expression analysis, 50 ng of RNA was used to perform SuperScript™ III first-strand synthesis, and miRNA expression was analyzed using Quanta-bioscience-IDT primers for mature miR-302a/b along with a universal control primer.

### Sno and SnRNA Real-Time PCR

50 ng of RNA was used to perform SuperScript™ III First-Strand Synthesis, and miRNA expression was analyzed using TaqMan assays for SNGH10, SNGH5, SNORD116a and RNU6ATAC according to the manufacturer’s instructions. A small nuclear RNA U6 primer was used as described^47^.

### Visualization and Statistical Analysis

The default setting of ArrayStar for Student t-test was used to determine significant transcripts. Direction of fold change from contrasts in multivariate analysis of variance (ANOVA) is used for comparison of miRNA microarray and miRNA sequencing for captured CD63^+^ and CD47^+^ EVs, and p < 0.05 is considered as significant. ArrayStar’s Scatter Plot was used for visual comparison of gene signals between captured vs uncaptured EVs replicated datasets. FDR < 0.1 (Benjamini-Hochberg) was applied to method I (Fig. S5). For the remaining analyses, unadjusted p < 0.05 was used.

### Data availability

Microarray data is deposited with accession GSE103310 at the Gene Expression Omnibus. RNAseq data is deposited on the NCBI Sequence Read Archive (GSE103493). Additional analysis of the sequencing data is available in the Supplementary Files.

## Electronic supplementary material

Supplemental data

Dataset 1

Dataset 2
