# Supplementary material for: CD63, MHC class 1, and CD47 identify subsets of extracellular vesicles containing distinct populations of noncoding RNAs
Source: Sci Rep. 2018 Feb 7;8:2577. doi: 10.1038/s41598-018-20936-7 (PMC5803193; doi:10.1038/s41598-018-20936-7)
Supplement: Supplementary file 1 — Supplemental data [file 41598_2018_20936_MOESM1_ESM.pdf]

Supplemental Data for

CD63, MHC class 1, and CD47 identify subsets of extracellular vesicles containing distinct populations of noncoding RNAs

Sukhbir Kaur[1], Abdel G. Elkahloun[2], Anush Arakelyan[3], Lynn Young[4], Timothy G. Myers[5], Francisco Otaizo-Carrasquero[5], Weiwei Wu[2], Leonid Margolis[3], and David D. Roberts[1]*

[1]Laboratory of Pathology, Center for Cancer Research, National Cancer Institute, National Institutes of Health, Bethesda MD 20982, USA

[2]Cancer Genetics Branch, National Human Genome Research Institute, National Institutes of Health, Bethesda, MD 20892, USA.

[3]Section of Intercellular Interactions, Eunice Kennedy-Shriver National Institute of Child Health and Human Development, National Institutes of Health, Bethesda MD 20982

[4]National Institutes of Health Library, Division of Library Services, Office of Research Services, National Institutes of Health, Bethesda, MD 20892, USA

[5]Genomic Technologies Section, Research Technologies Branch, National Institute of Allergy and Infectious Diseases, National Institutes of Health, Bethesda, MD 20892, USA

Supplementary Figure Legends

Figure S1: (a, b) Capturing efficiency of CD63 conjugated MNPs. **(c-d)** Extracted EVs were prepared using the Exo-Quick kit, and vesicle size and concentration were measured using Nanosight.

Figure S2: (a) Side scattering for calibrating beads of the indicated sizes. **(b)** Bodipy-FL labeled EVs released from Jurkat cells were captured with anti-CD63-MNPs and analyzed by flow cytometry. Volumetric control was used to estimate the concentration of captured EVs

Figure S3: (a) Bodipy labeled EVs released from Jurkat or JinB8 cells where captured with anti-MHC1-MNPs and analyzed using flow cytometry. Volumetric control was used to estimate the concentration of captured EVs

Figure S4: RNA profiles were obtained using a RNA Bioanalyzer for the indicated capture vs uncaptured CD47, CD63 and MHC1-EVs for RNA sequencing samples.

Figure S5: Experimental design and work flow for the RNA-sequencing data analysis of selected small RNAs.

Figure S6: (a-c) RNA profiles were obtained using RPKM normalization comparing captured and uncaptured CD47, CD63 and MHC1 EVs with absolute value of the fold change greater that 2.0 using scale log base 2. The number of upregulated RNAs in captured CD47, CD63 and MHC1 EVs are shown as pie charts.

Figure S7: (a) Scatter graph of biological replicates comparing CD47^{+} and CD63^{+} EVs showing 24 transcripts ($p < 0.05$ and absolute value of the fold change greater that 2.0) using scale log base 2 of total RPM, (CD47_CAP and CD63_CAP, Student's- t-test). 627 transcripts pass the fold change cutoff before the application of the statistical test. **(b)** A Scatter graph of biological replicates (n=2) between CD47^{+}/MHC1^{+} EVs showing 682 transcripts at 2-fold change before application of the statistical test and 41 (White) at $p < 0.05$ using log base 2 of total RPM, X-axis (CD47_CAP and MHC1_CAP). **(c)** A Scatter graph of biological replicates comparing CD63^{+} and MHC1^{+} EVs showing 483 transcripts at 2-fold change before application of the statistical test and 9 highlighted (White) at $p < 0.05$ using scale log base 2 of total RPM, (CD63_CAP and MHC_CAP). **(d)** Heat map of hierarchical clustering of the 24 transcripts at $p<0.05$ comparing CD47^{+} and CD63^{+} EVs. (**e)** Heat map of hierarchical clustering of the 38 transcripts at $p<0.05$ comparing CD63^{+}

and CD63^{-} EVs. **(f)** Heat map of hierarchical clustering of the 9 transcripts at $p<0.05$ comparing CD63^{+} and MHC1^{+} EVs.

FigureS8: (a) A Scatter graph of biological replicates (n=3) between CD47^{+}/CD47^{-} EVs showing 109 miRNA (All species) highlighted (White) at $p<0.05$ using scale log base 2 of total RPM, (CD47_CAP and CD47_UnCap). **(b)** A Scatter graph of biological replicates (n=2) comparing CD63^{+} and CD63^{-} EVs showing 208 miRNAs (All species) highlighted (White) at $p<0.05$ using scale log base 2 of total RPM, (CD63_CAP and CD63_UnCap). (**c)** A Scatter graph of biological replicates (n=2) between MHC1^{+}/MHC1^{-} EVs showing 135 miRNAs highlighted (White) at $p<0.05$ using scale log base 2 of total RPM, X-axis (MHC1_CAP and MHC_UnCap).

Figure S9: (a) Hierarchical clustering of 135 miRNAs (All species) that were significant at $p<0.05$ (Student-t test) comparing MHC1^{+} AND MHC^{-} EVs. **(b)** Out of 135 miRNAs, 7 hsa-miRNA were enriched in MHC1^{+} EVs. A comparison of MHC1^{+} and CD47^{+} EVs showed differential expression of 5 hsa-miRNAs. **(c)** Microarray ratio files were imported into miRNA analysis Array Star project, and fold change was calculated by comparing CD47^{+} and CD63^{+} EVs and CD47^{+b} and CD63^{+b} EVs. Using the 1.5 fold change cutoff and P-value ≤ 0.05, 60 miRNA were found common between the RNA sequencing and microarray analyses. The pie chart shows UP (upregulated in CD47^{+} and CD63^{+} EVs), Down (in both), and UP/DOWN (not consistent). **(d)** Venn diagram of Captured CD63^{+}, CD47^{+} and MHC1^{+} EV miRNAs identified by sequencing analysis aligned with the non-coding genome using DNA Array Star 14.

Figure S1

CD63 capture efficiency

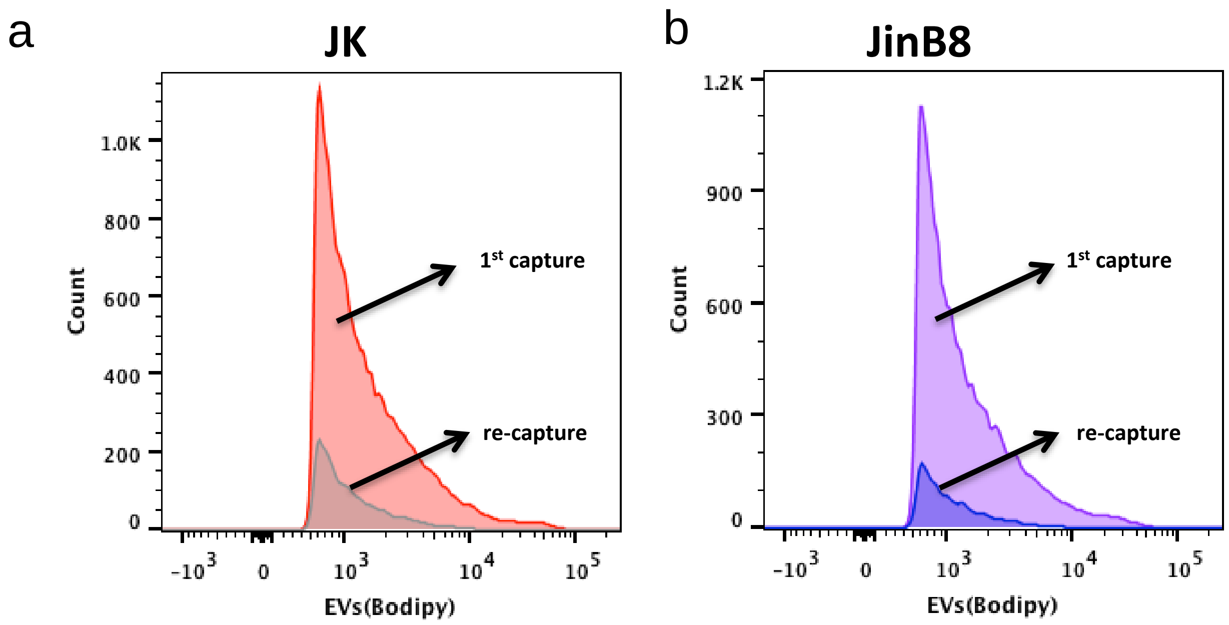

83.1% **87.8%**

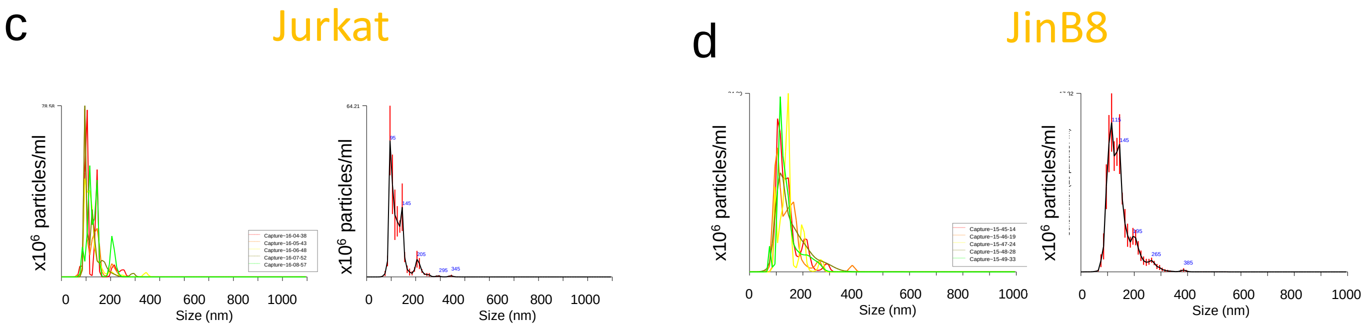

Mean: 122+/-3.0nm
Mode: 101.6 +/-3.7nm

Mean: 140.1+/-2.8nm
Mode: 117.7 +/-6.8nm

Figure S2

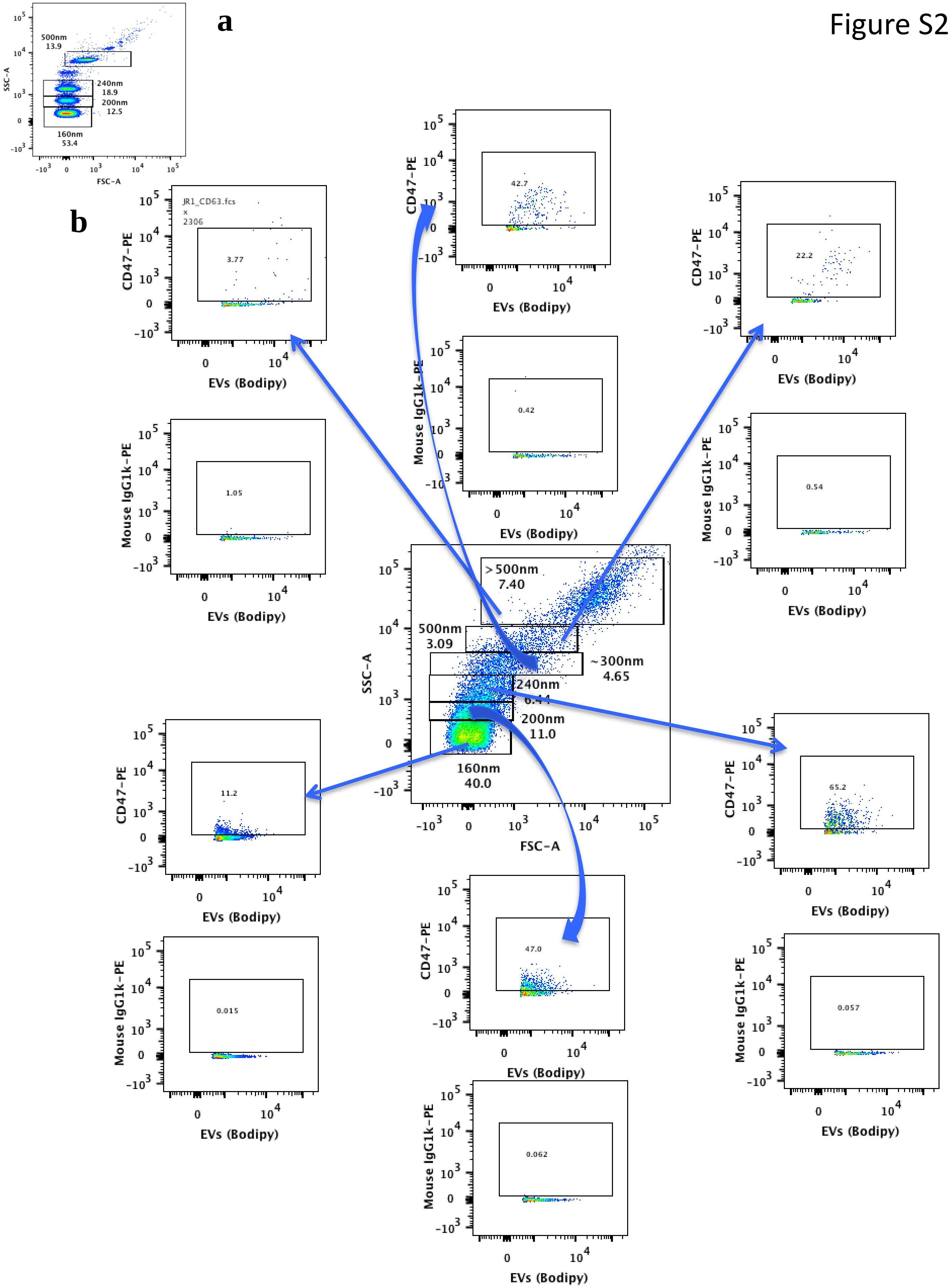
a
500nm
13.9
240nm
18.9
200nm
12.5
160nm
53.4
SSC-A
FSC-A
b
JR1_CD63.fcs
2306
3.77
42.7
22.2
1.05
0.42
0.54
11.2
47.0
65.2
0.015
0.062
0.057
CD47-PE
Mouse IgG1k-PE
EVs (Bodipy)
>500nm
7.40
500nm
3.09
~300nm
4.65
240nm
200nm
11.0
160nm
40.0

Figure S3

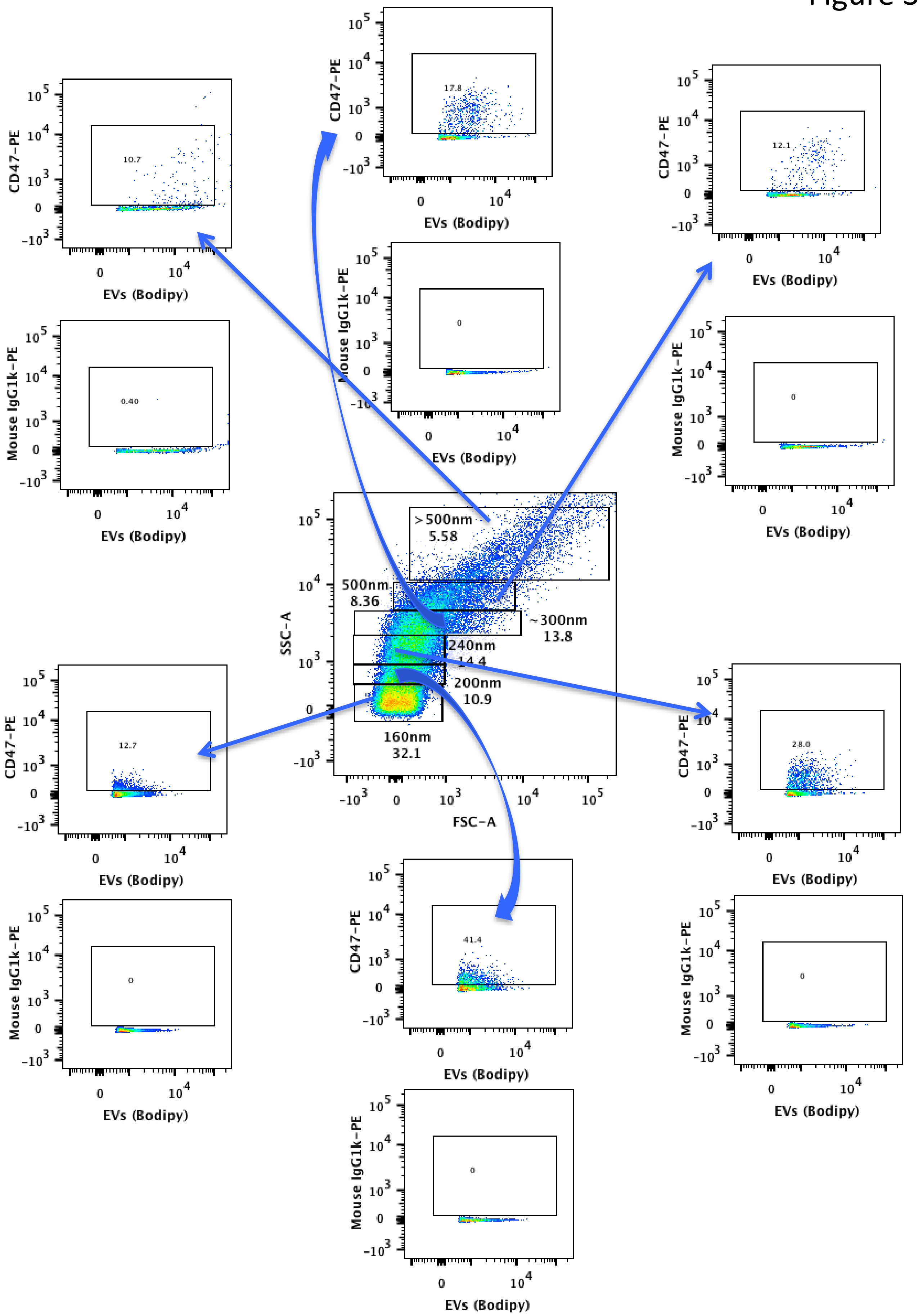

CD47-PE
Mouse IgG1k-PE
EVs (Bodipy)
SSC-A
FSC-A
10.7
17.8
12.1
0.40
0
12.7
28.0
41.4
>500nm
5.58
500nm
8.36
~300nm
13.8
240nm
14.4
200nm
10.9
160nm
32.1

Figure S4

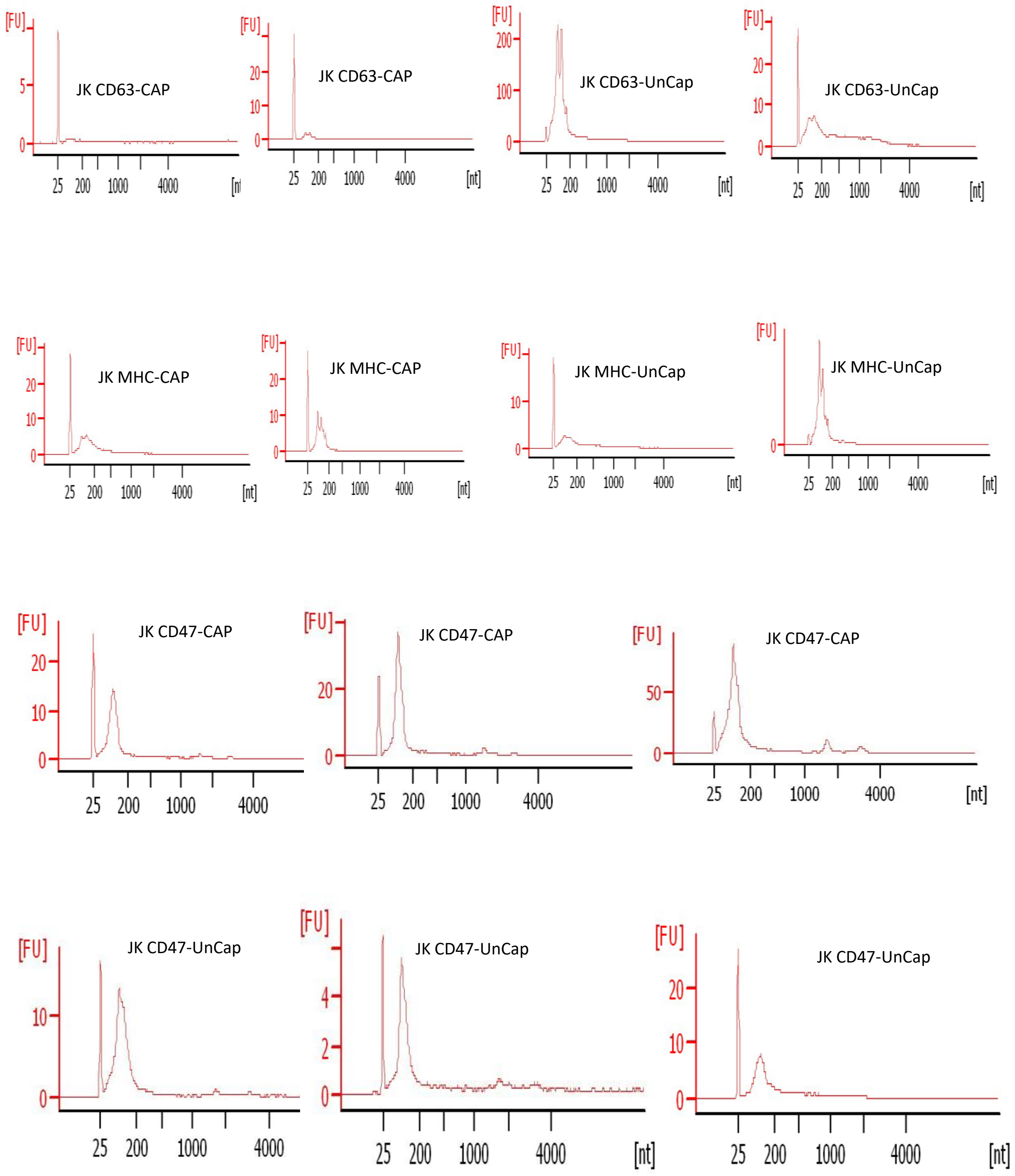
JK CD63-CAP
JK CD63-CAP
JK CD63-UnCap
JK CD63-UnCap
JK MHC-CAP
JK MHC-CAP
JK MHC-UnCap
JK MHC-UnCap
JK CD47-CAP
JK CD47-CAP
JK CD47-CAP
JK CD47-UnCap
JK CD47-UnCap
JK CD47-UnCap
[FU]
[nt]
25
200
1000
4000

Figure S5

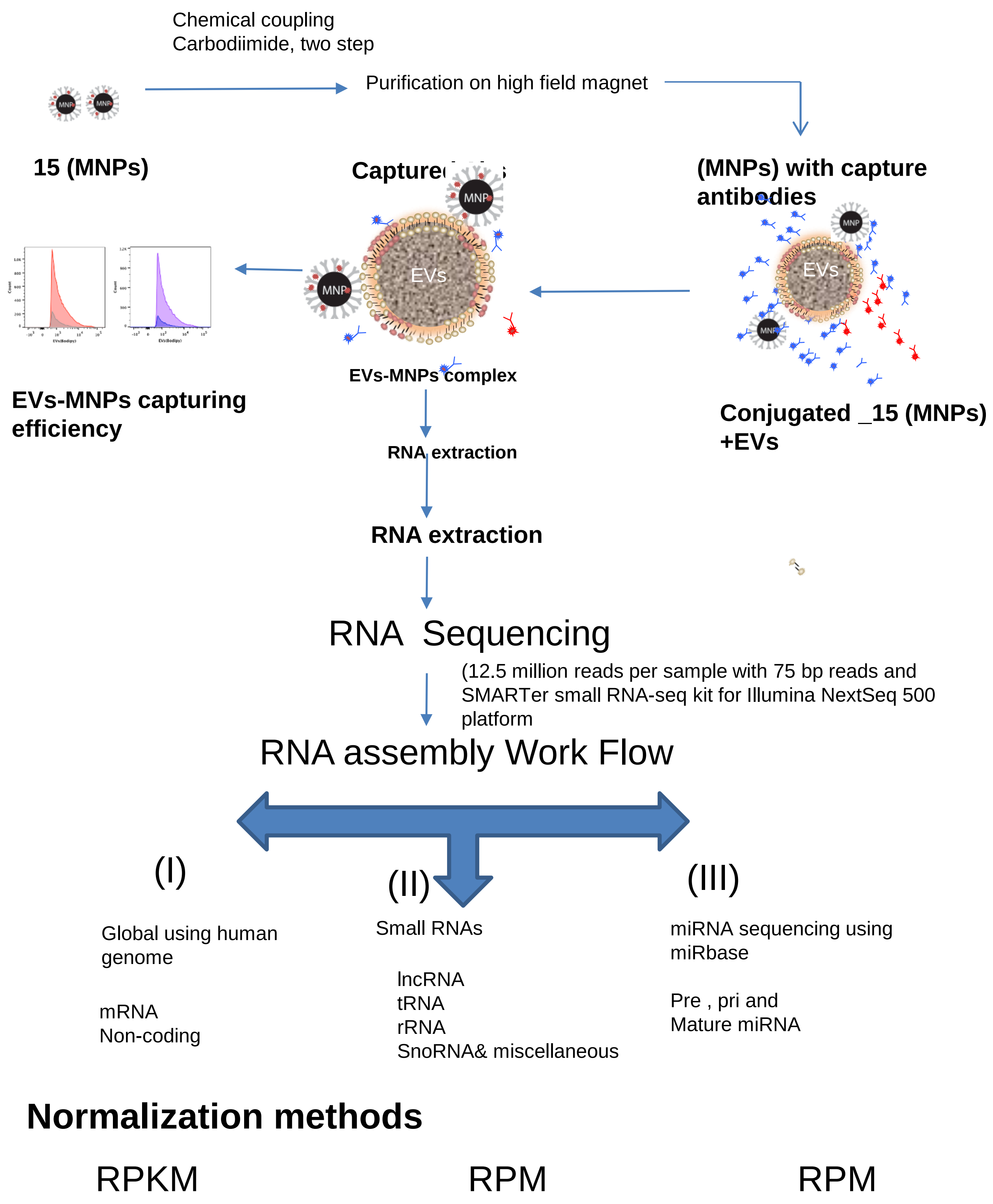
Chemical coupling
Carbodiimide, two step
Purification on high field magnet
MNP
15 (MNPs)
Capture
MNP
EVs
EVs-MNPs complex
(MNPs) with capture antibodies
MNP
EVs
Conjugated _15 (MNPs) +EVs
EVs-MNPs capturing efficiency
RNA extraction
RNA extraction
RNA Sequencing
(12.5 million reads per sample with 75 bp reads and SMARTer small RNA-seq kit for Illumina NextSeq 500 platform
RNA assembly Work Flow
(I)
Global using human genome
mRNA
Non-coding
(II)
Small RNAs
lncRNA
tRNA
rRNA
SnoRNA& miscellaneous
(III)
miRNA sequencing using miRbase
Pre , pri and
Mature miRNA
Normalization methods
RPKM
RPM
RPM

Figure S6

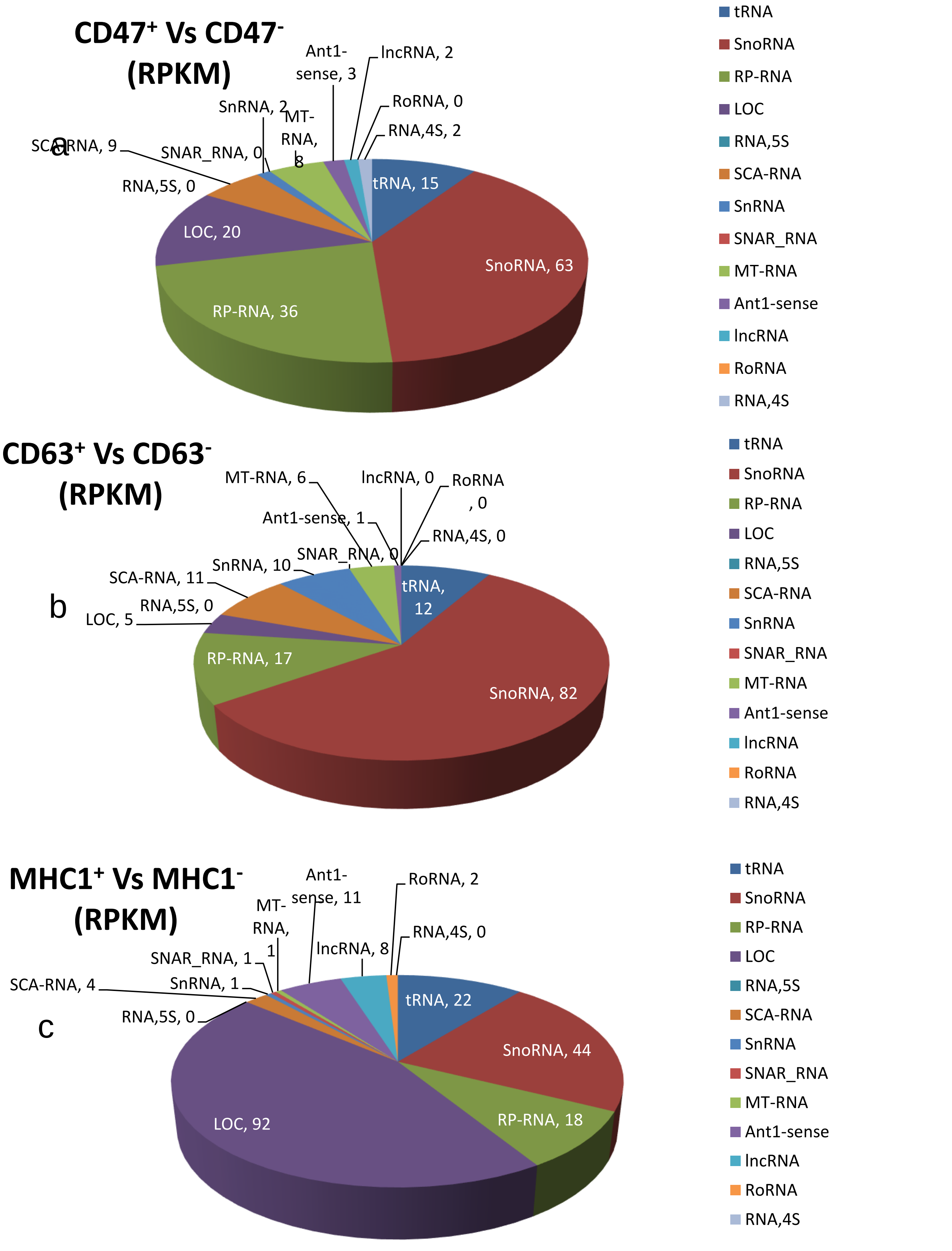

CD47+ Vs CD47- (RPKM)
a
tRNA, 15
SnoRNA, 63
RP-RNA, 36
LOC, 20
RNA,5S, 0
SCA-RNA, 9
SNAR_RNA, 0
SnRNA, 2
MT-RNA, 8
Ant1-sense, 3
lncRNA, 2
RoRNA, 0
RNA,4S, 2
tRNA
SnoRNA
RP-RNA
LOC
RNA,5S
SCA-RNA
SnRNA
SNAR_RNA
MT-RNA
Ant1-sense
lncRNA
RoRNA
RNA,4S
CD63+ Vs CD63- (RPKM)
b
MT-RNA, 6
lncRNA, 0
RoRNA, 0
Ant1-sense, 1
RNA,4S, 0
SNAR_RNA, 0
SnRNA, 10
SCA-RNA, 11
RNA,5S, 0
LOC, 5
tRNA, 12
RP-RNA, 17
SnoRNA, 82
tRNA
SnoRNA
RP-RNA
LOC
RNA,5S
SCA-RNA
SnRNA
SNAR_RNA
MT-RNA
Ant1-sense
lncRNA
RoRNA
RNA,4S
MHC1+ Vs MHC1- (RPKM)
c
Ant1-sense, 11
RoRNA, 2
MT-RNA, 1
RNA,4S, 0
lncRNA, 8
SNAR_RNA, 1
SCA-RNA, 4
SnRNA, 1
RNA,5S, 0
tRNA, 22
SnoRNA, 44
RP-RNA, 18
LOC, 92
tRNA
SnoRNA
RP-RNA
LOC
RNA,5S
SCA-RNA
SnRNA
SNAR_RNA
MT-RNA
Ant1-sense
lncRNA
RoRNA
RNA,4S

Figure S7

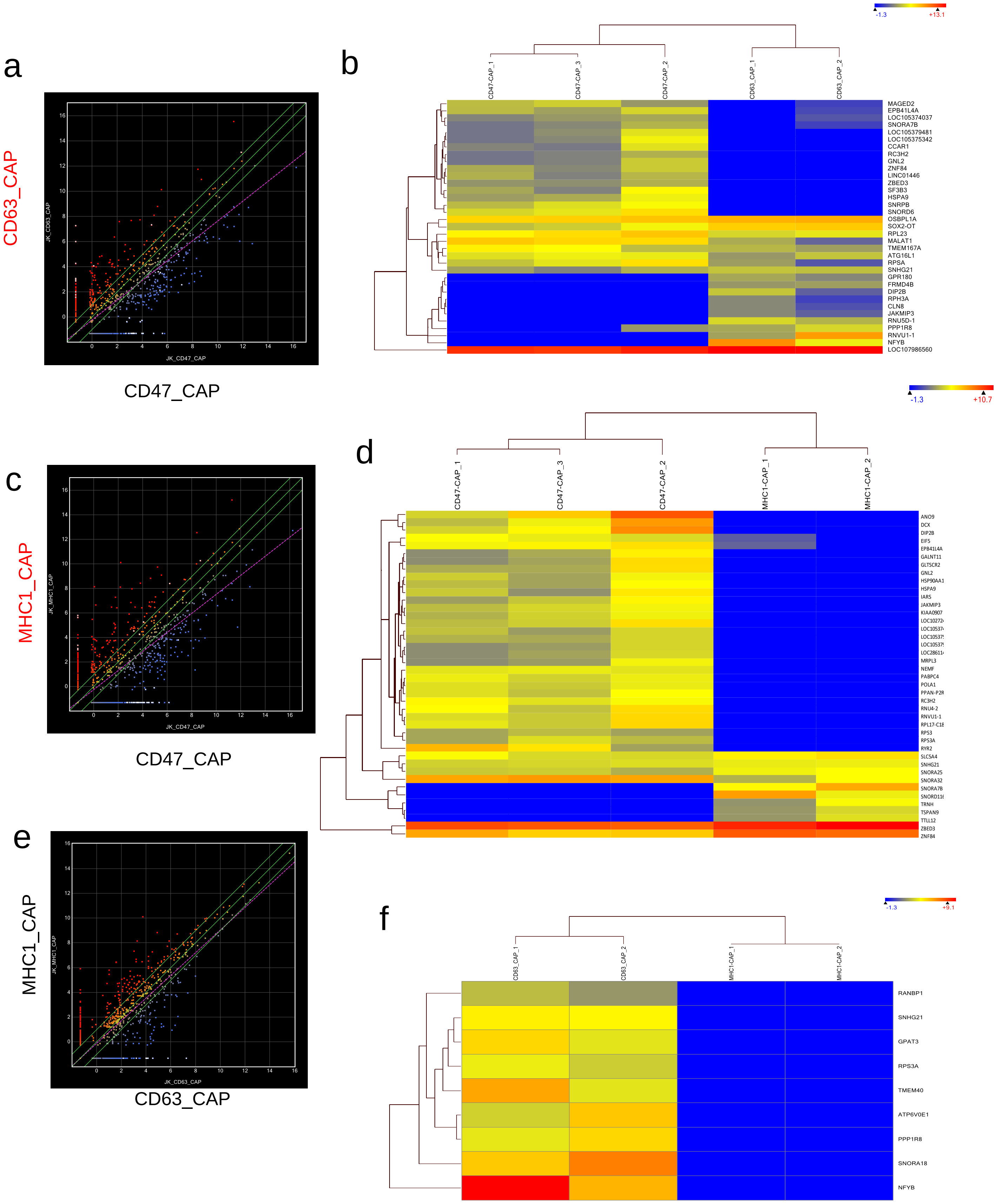

a
CD63_CAP
JK_CD63_CAP
JK_CD47_CAP
CD47_CAP
b
-1.3
+13.1
CD47-CAP_1
CD47-CAP_3
CD47-CAP_2
CD63_CAP_1
CD63_CAP_2
MAGED2
EPB41L4A
LOC105374037
SNORA7B
LOC105379481
LOC105375342
CCAR1
RC3H2
GNL2
ZNF84
LINC01446
ZBED3
SF3B3
HSPA9
SNRPB
SNORD6
OSBPL1A
SOX2-OT
RPL23
MALAT1
TMEM167A
ATG16L1
RPSA
SNHG21
GPR180
FRMD4B
DIP2B
RPH3A
CLN8
JAKMIP3
RNU5D-1
PPP1R8
RNVU1-1
NFYB
LOC107986560
c
MHC1_CAP
JK_MHC1_CAP
JK_CD47_CAP
CD47_CAP
d
-1.3
+10.7
CD47-CAP_1
CD47-CAP_3
CD47-CAP_2
MHC1-CAP_1
MHC1-CAP_2
ANO9
DCX
DIP2B
EIF5
EPB41L4A
GALNT11
GLTSCR2
GNL2
HSP90AA1
HSPA9
IARS
JAKMIP3
KIAA0907
MRPL3
NEMF
PABPC4
POLA1
RC3H2
RNU4-2
RNVU1-1
RPS3
RPS3A
RYR2
SLC5A4
SNHG21
SNORA25
SNORA32
SNORA7B
TRNH
TSPAN9
TTLL12
ZBED3
ZNF84
e
MHC1_CAP
JK_MHC1_CAP
JK_CD63_CAP
CD63_CAP
f
-1.3
+9.1
CD63_CAP_1
CD63_CAP_2
MHC1-CAP_1
MHC1-CAP_2
RANBP1
SNHG21
GPAT3
RPS3A
TMEM40
ATP6V0E1
PPP1R8
SNORA18
NFYB

Figure S8

Method III

a

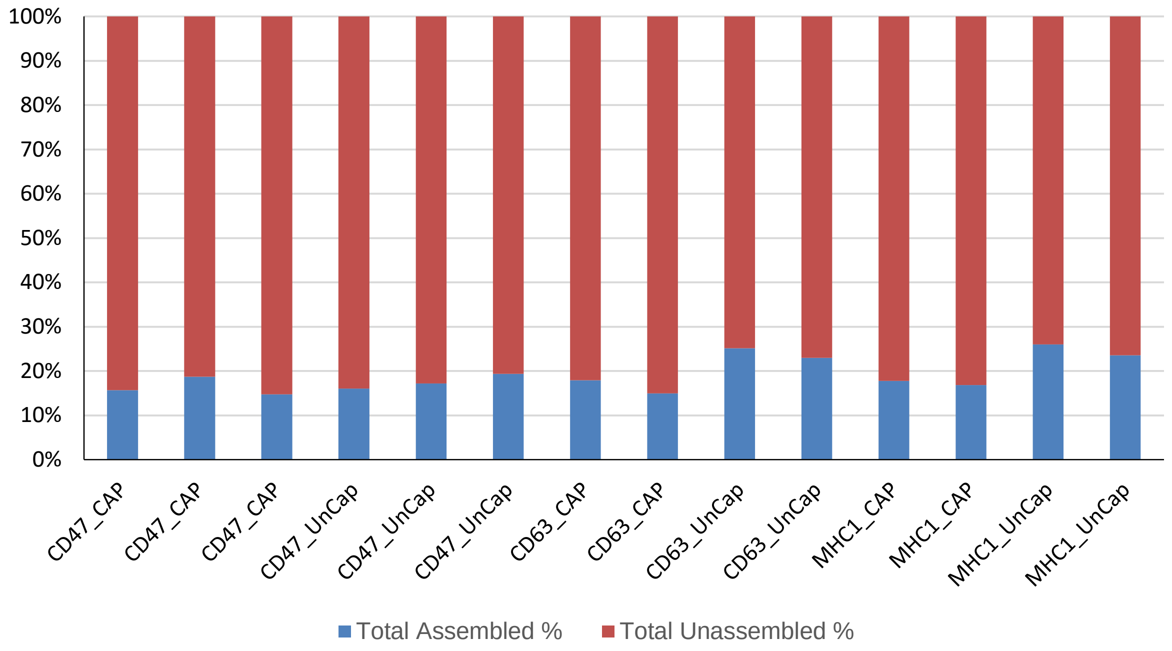

b

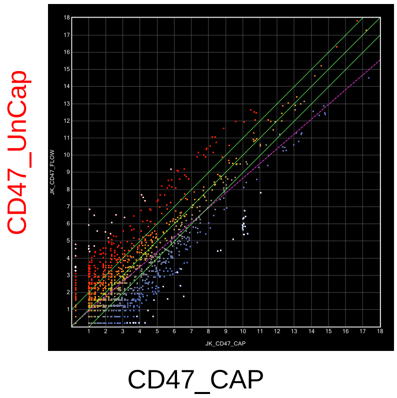

c

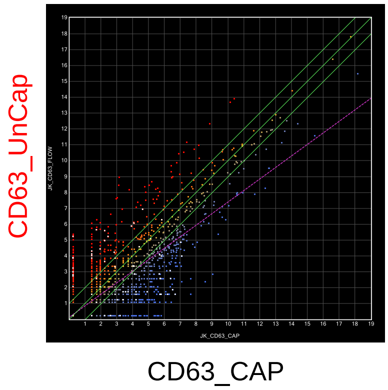

d

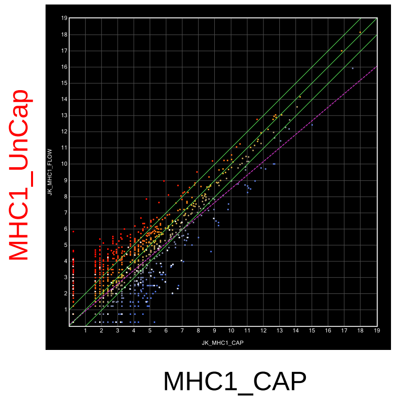

66miRNA @8-fold
1491miRNAs@2-fold

240miRNAs @8-fold
1971miRNAs@2-fold

97miRNAs @8-fold
1768miRNAs@2-fold

Figure S9

a

	CD47_CAP - linear total RPM	CD63_CAP - linear total RPM	MHC1_CAP - linear total RPM
hsa-mir-106b	1.179	1.179	9.571
hsa-miR-142-3p	2.619	10.64	9.571
hsa-miR-185-5p	1.179	1.179	9.571
hsa-mir-3610	2.238	1.179	9.571
hsa-mir-620	1.179	1.179	9.571
hsa-mir-663b	7.304	2.718	9.571
hsa-mir-664b	21.369	4.616	9.571

b

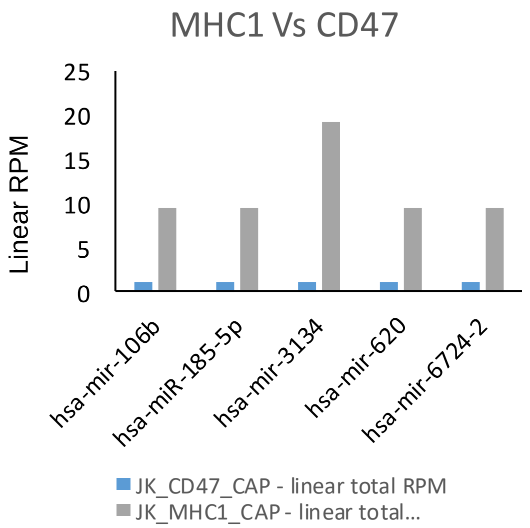

c

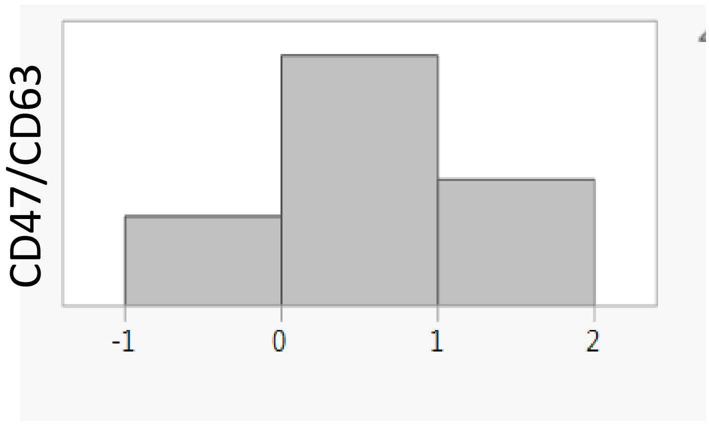

d

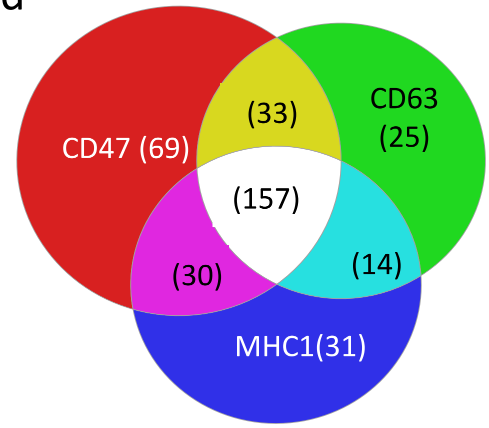

Long non-coding +small RNAs

Supplementary Excel file notes for Kaur et al

File Name: Table A _B_new Fig2_ Method I_RPKM

Contents

1. Data for Figure 2 pie charts (21 excel sheets) -Table-A
2. Data for FDR analysis with RPKM_Method (I)-Table B
3. Table S3 (Mapped and unmapped reads using Method (1)
4. Fig S6 2-fold change Captured vs uncaptured

File Name: Table S4_ Raw data

Contents

1. Table S4 (Mapped and unmapped reads using Method (III)
